# Orthogonal spectroscopic assessment of higher order structure of nucleic acids and its concentration-dependent polymorphism

**DOI:** 10.1016/j.omtn.2026.103131

**Published:** 2026-10-07

**Authors:** Taiji Oyama, Satoko Suzuki, Ken-ichi Akao, Kazunori Ikebukuro, Kohsaku Kawakami, Tomohiko Yamazaki

**Affiliations:** 1 JASCO Corporation, Ishikawa-machi, Tokyo 192-8537, Japan; 2 Department of Biotechnology and Life Science, Tokyo University of Agriculture and Technology, 2-24-16 Naka-cho, Koganei, Tokyo 184-8588, Japan; 3 Research Center for Macromolecules and Biomaterials (RCMB), National Institute for Materials Science (NIMS), 1-2-1, Sengen, Tsukuba 305-0047, Japan; 4 Division of Life Science, Hokkaido University, Kita 10, Nishi 8, Kita-ku, Sapporo 060-0808, Japan

**Keywords:** MT: Oligonucleotides: Therapies and Applications, circular dichroism spectroscopy, Raman spectroscopy, infrared spectroscopy, orthogonal spectroscopy, higher order structure, nucleic acid therapeutics, concentration-dependent structural polymorphism, pharmaceutical formulation

## Abstract

Nucleic acids can adopt multiple higher order structures (HOSs) from a single sequence, and such structural polymorphism may change in a concentration-dependent manner. This suggests that nucleic acid therapeutics may undergo concentration-dependent structural changes under formulation-relevant conditions. However, systematic evaluation of this behavior has been limited because spectroscopic techniques are typically applied over different concentration ranges. Here, we propose an orthogonal spectroscopic assessment strategy that enables circular dichroism (CD), Raman, and Fourier transform infrared (FT-IR) spectroscopy to be performed under closely matched concentration conditions. Using the thrombin-binding aptamers, we investigated concentration-dependent changes in HOS over a formulation-relevant concentration range. CD combined with reference-based principal component analysis revealed distinct concentration-dependent structural transitions. Complementary Raman and FT-IR analyses provided insights into nucleobase orientation, hydrogen-bonding environments, and phosphate backbone conformations, supporting the CD-based interpretations. Notably, one sequence showed G-quadruplex (G4)-like spectroscopic features associated with higher order assembly at high concentrations, despite its low predicted G4-forming propensity based on sequence information alone. These results indicate that concentration-dependent structural polymorphism should be evaluated in the characterization of nucleic acid therapeutics. The strategy presented here provides a practical platform for evaluating HOS under formulation-relevant conditions and supports the development and quality assessment of nucleic acid therapeutics.

## Graphical abstract

**Figure:**
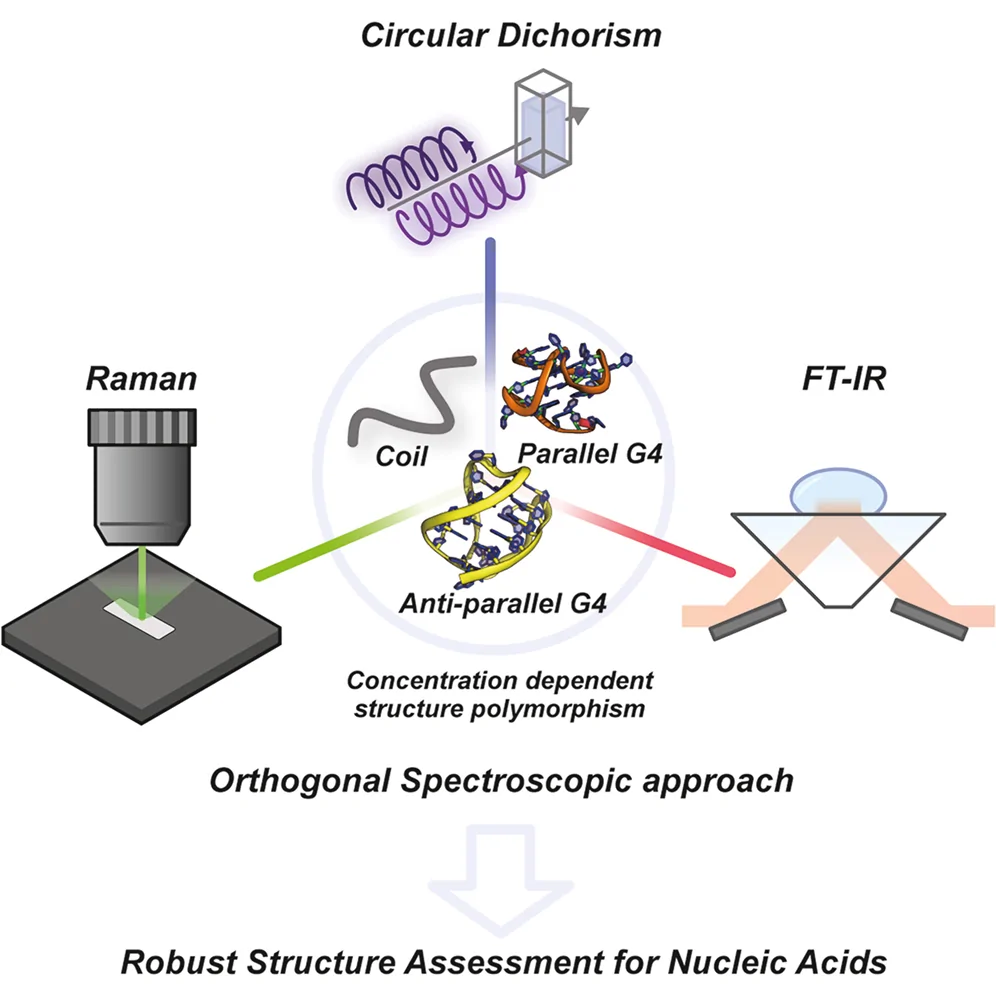

## Teaser

Yamazaki and colleagues demonstrated that an orthogonal spectroscopic strategy, utilizing circular dichroism (CD), Raman, and Fourier-transform infrared (FT-IR) analyses, enables systematic evaluation of concentration-dependent higher order structure changes in nucleic acid therapeutics. This provides a practical platform for the characterization and quality assessment of nucleic acid-based drugs relevant to formulation.

## Introduction

Nucleic acid therapeutics are pharmaceutical agents in which an oligonucleotide composed of several to several tens of nucleotides serves as the active ingredient. These molecules regulate biological function by directly binding to their target RNA or protein, without requiring modulation of gene-expression processes.^1^ With the therapeutic targets for small-molecule and antibody drugs becoming increasingly depleted, nucleic acid therapeutics are gaining attention for their ability to act through mechanisms distinct from these conventional modalities. In addition, unlike antibody therapeutics that require cell-culture-based manufacturing processes, nucleic acid therapeutics can be produced by chemical synthesis, offering advantages in batch uniformity and structural tunability.^2^ These features have enabled nucleic acid therapeutics to rapidly emerge as a new modality following small-molecule and antibody drugs. Since the approval of fomivirsen by the US Food and Drug Administration in 1998, advances in chemical modification technologies and drug-delivery systems have accelerated their development,^2^ and more than 20 nucleic acid therapeutics have been approved in the United States, Europe, or Japan as of 2025. Their therapeutic indications span a wide range of diseases, from ophthalmic disorders to muscular and metabolic diseases and amyloidosis-related conditions. The nucleic acid therapeutics approved to date can be broadly classified into four categories: antisense oligonucleotides,^3^ small interfering RNA (siRNA),^4^ aptamers,^5^ and CpG oligodeoxynucleotides.^6^

The importance of higher order structure (HOS) is evident for aptamers, whose activity depends on adopting specific three-dimensional folding, and for siRNA, which requires formation of a double-stranded structure. Although antisense oligonucleotides act through sequence-dependent duplex formation with complementary mRNA and may therefore appear less dependent on the HOS, a study has reported that intramolecular folding can diminish their *in vivo* activity.^7^^,^^8^^,^^9^

Because the injection volume for subcutaneously administered formulations is typically limited to less than 2 mL,^10^^,^^11^ many nucleic acid therapeutics are formulated at concentrations exceeding 100 mg/mL. In antibody therapeutics, aggregation is regarded as one of the factors underlying immunogenicity, and consequently, substantial efforts have been directed toward understanding concentration-dependent changes in the HOS and aggregation under high-concentration formulation conditions.^12^^,^^13^^,^^14^^,^^15^^,^^16^

It has been reported that nucleic acids can exhibit structural polymorphism within a single sequence.^17^ Recent studies have demonstrated that multiple conformations can coexist in equilibrium.^18^^,^^19^ Such equilibria are expected to shift in a concentration-dependent manner. Indeed, telomeric G-quadruplexes (G4s) have been reported to undergo concentration-dependent changes in their topology.^20^^,^^21^ Conducting similar assessments for nucleic acid therapeutics is expected to support the development of safer and more reliable drug products. We previously reported that, in experiments using model oligonucleotides derived from the thrombin-binding aptamer (TBA) sequence, some oligonucleotide solutions exhibited concentration-dependent changes in solubility, viscosity, and liquid-liquid phase separation (LLPS).^22^ In addition, it has been reported that a morpholino-modified oligonucleotide showed reduced activity owing to aggregation in solution.^23^ These observations indicate that evaluating the HOS of nucleic acids across a range of concentrations is essential for pharmaceutical development.

Spectroscopic techniques constitute one approach for evaluating the HOS of nucleic acids. Circular dichroism (CD) spectroscopy is widely used to evaluate DNA HOSs because it is sensitive to base orientation, base stacking, and base pairing. This technique has been applied not only to canonical DNA structures, such as duplex and single-stranded conformations, but also to non-canonical structures, including G4 structures, whose characteristic spectral patterns are often used to infer folding topology.^17^^,^^24^^,^^25^^,^^26^^,^^27^^,^^28^^,^^29^^,^^30^^,^^31^ Infrared spectroscopy and Raman spectroscopy provide complementary structural information derived from the phosphate backbone, nucleobases, and intermolecular interactions, thereby allowing assessment of the HOS.^21^^,^^32^^,^^33^^,^^34^^,^^35^ Although spectroscopic techniques do not provide the high-resolution structural information that can be obtained using X-ray crystallography or nuclear magnetic resonance (NMR) spectroscopy, they offer practical advantages in that measurements are straightforward, and data can be acquired within minutes for each sample. These features make them useful for quality control and comprehensive characterization of pharmaceutical products.^36^^,^^37^^,^^38^

For the evaluation of pharmaceutical products, it is desirable to assess each critical quality attribute using an orthogonal assessment strategy, in which multiple analytical methods based on different measurement principles are employed.^39^ In the case of antibody therapeutics, combinations of CD, infrared, and Raman spectroscopy are commonly used for this purpose.^36^^,^^37^^,^^40^^,^^41^^,^^42^

Although CD, Raman, and infrared spectroscopy have each been applied to characterize nucleic acid structures, studies that systematically compare these techniques using the same nucleic acid samples under formulation-relevant concentration conditions are still limited. This gap largely arises because CD and vibrational spectroscopies are typically applied over different and non-overlapping concentration ranges. CD spectroscopy is typically performed at sample concentrations around 0.1 mg/mL, whereas infrared and Raman spectroscopy measurements are generally conducted at concentrations above 10 mg/mL. In CD spectroscopy, the use of an ultrashort-path-length cell enables measurement of proteins and nucleic acids at high concentrations. Vanloon et al. demonstrated that CD spectra of DNA at concentrations of approximately 1 mM can be obtained by reducing the optical path length.^43^ Using CD spectroscopy combined with a short-path-length cell, Renčiuk et al. reported that the topology of telomeric G4 changes at concentrations above 40 mM.^20^ Using Raman spectroscopy and CD spectroscopy with a short-path-length cell, Palacký et al. further showed that the telomeric G4 topology changes in a concentration-dependent manner at concentrations above 8 mM.^21^ Evaluating the practical applicability of ultrashort-path-length cells for the characterization of formulated nucleic acid therapeutics and establishing a systematic orthogonal assessment by extending CD measurements into the concentration range used for Raman and Fourier transform infrared (FT-IR) spectroscopy would be valuable for formulation research.

In the present study, TBA and modified TBAs were selected as model DNA oligonucleotides based on our previous study (Table 1).^22^ In that study, these sequences were designed from the known G4-forming TBA scaffold to provide a set of guanine-rich DNA oligonucleotides with similar sequences but distinct G4-related structural features. The model sequences were shown to adopt a different HOS, and these structural differences were associated with formulation-relevant physicochemical properties, including solubility, viscosity, and LLPS. In particular, one modified sequence, referred to as T4, which did not show clear evidence of a stable HOS under diluted to moderately concentrated conditions, exhibited LLPS and markedly high non-Newtonian viscosity at higher concentrations than the G4-forming sequences. These observations led to the hypothesis that the absence of a compact HOS results in an elongated molecular state, which enables entanglement of the oligonucleotide chains. However, structural characterization in the previous study was mainly performed under dilute or moderately concentrated conditions, and concentration-dependent HOS changes under highly concentrated conditions were not directly examined. In this study, we aimed to establish an orthogonal spectroscopic approach that enables CD, attenuated total reflection (ATR) FT-IR, and Raman spectroscopy to be applied to samples prepared under closely matched buffer and overlapping, formulation-relevant concentration conditions. Using this approach, we demonstrated that the HOS of the model TBAs undergoes concentration-dependent changes.

**Table 1:** Information for TBA and modified TBAs used in this study

Name	Sequence	Sequence modification/design	Expected structure from previous report^22^	Number of bases	G-score
TBA	GGTTGGTGTGGTTGG	parent thrombin-binding aptamer sequence	antiparallel G4	15	20
T1	GGGTTGGGTGTGGGTTGGG	G-tracts were extended from two to three consecutive guanines	stable G4; parallel or hybrid depending on buffer	19	41
T2	GGGTTGGTGTGGTTGGG	some G-tracts were extended, resulting in unmatched G-tract lengths	antiparallel G4 with possible structural heterogeneity	17	21
T3	GGGTTGGTGTGGTTGGTGGG	an additional G-rich segment was introduced	antiparallel G4 with dimer formation	20	20
T4	GTGTGGTGTGGTGTG	G-tract continuity was disrupted while maintaining similar GC content	no clear G4-like CD signals; no obvious UV melting transition	15	–

All oligonucleotides examined in this study were composed of deoxyribonucleotides. The G-scores were calculated using QGRS Mapper (https://bioinformatics.ramapo.edu/QGRS/index.php).^44^

## Results

### CD spectra of nucleic acids

Figure 1 shows CD spectra of TBA and modified TBAs. At a concentration of 0.1 mg/mL, the CD spectrum of TBA exhibits a positive band at around 250 nm, a negative band at around 265 nm, and a positive band at around 295 nm, indicating the formation of a typical antiparallel G4 (Figure 1A). The shape of the TBA spectrum is retained at higher concentrations, suggesting no substantial changes in its HOS. For T1 at a concentration of 0.1 mg/mL, a small negative band appears at around 242 nm and a positive band at around 262 nm, which is consistent with a parallel G4 topology (Figure 1B). A shoulder at around 290 nm is observed, and its intensity decreases above 5 mg/mL.

**Figure 1:**
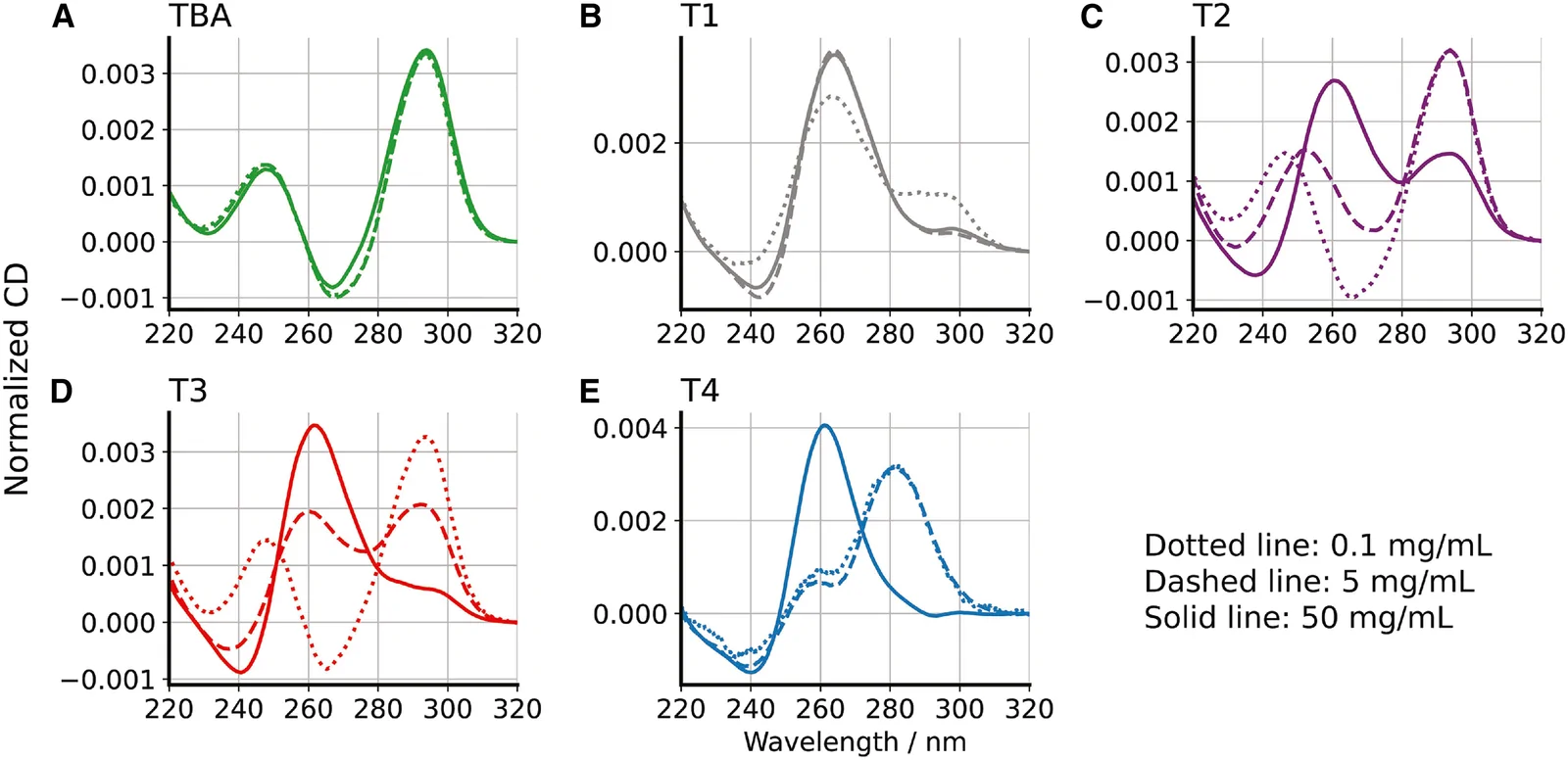
CD spectra of TBA and modified TBAs (A) TBA, (B) T1, (C) T2, (D) T3, and (E) T4. Spectra recorded at 0.1, 5, and 50 mg/mL are shown as dotted, dashed, and solid lines, respectively. Each spectrum represents the mean of three measurements.

T2 and T3 exhibit clear concentration-dependent changes in their CD spectra. At a concentration of 0.1 mg/mL, both display antiparallel-type CD signatures similar to those for TBA. For T2, no marked change is observed in the intensity of the positive band at around 295 nm at 5 mg/mL, whereas the intensity around 250 nm decreases and that of the positive band at around 262 nm increases (Figure 1C). At 50 mg/mL, the positive band at around 262 nm further increases, while the intensities around 250 and 295 nm decrease, indicating a shift toward a more parallel-like conformation. For T3, the intensities around 250 nm and at the positive band at around 295 nm decrease at 5 mg/mL, whereas the intensity for the positive band at around 262 nm increases (Figure 1D). These changes become more pronounced at 50 mg/mL, indicating a shift toward a more parallel-like conformation, similar to that observed for T2.

In contrast, T4 shows no CD features characteristic of G4 at a concentration of either 0.1 or 5 mg/mL, suggesting a non-G4 conformation (Figure 1E). The CD spectrum of T4 does not fully coincide with that measured at 95°C, which is assumed to represent the unfolded state (Figure S1). This observation suggests that T4 may not adopt a fully random-coil conformation and may instead form another type of structure. The G4-forming potential of TBA and each modified TBA was further evaluated using thermal difference spectra (TDSs) (Figure S2). TBA, T1, T2, and T3 at 0.1 mg/mL show characteristic TDS patterns associated with G4 formation, with positive peaks at approximately 243 and 273 nm and a negative peak at approximately 295 nm.^45^ In contrast, T4 does not show characteristic double-positive peaks at 243 and 273 nm at 0.1 mg/mL. This observation is consistent with the G-score calculated using QGRS Mapper,^44^ which provides a sequence-based prediction score for G4-forming potential. T4 does not yield a G-score, whereas the other sequences give G-scores of 20 or higher, supporting their predicted propensity to form G4 structures (Table 1). At 50 mg/mL, T4 displays a negative CD band at around 242 nm and a positive band at around 262 nm, which is consistent with the possible formation of a parallel G4-like structure (Figure 1E).

Thioflavin T (ThT) fluorescence measurements were also performed as an additional supportive assay for G4-like structural features (Figure S3). At 0.1 mg/mL, T4 shows the lowest ThT fluorescence intensity among TBA and the modified TBAs, consistent with the CD and TDS results (Figures 1E and S2E). In addition, a G-score analysis indicates that T4 is unlikely to form a G4 structure under these conditions. In contrast, at 50 mg/mL, T4 exhibits the highest relative ThT fluorescence intensity among all TBA and modified TBAs. These results are consistent with the concentration-dependent changes observed in the CD spectra and provide additional support for the presence of G4-like structural features in T4 at high concentration. However, because the ThT assay does not directly assess molecular structure, the possibility that factors other than G4 formation contribute to the enhanced ThT fluorescence cannot be completely excluded.

Because T4 contains only two short G-tracts, each with no more than two consecutive guanines, it is unlikely to form an intramolecular G4. In addition, T4 exhibits markedly higher viscosity (41.3 mPa s) than T1 (1.9 mPa s) at 100 mg/mL in water.^22^ These observations raise the possibility that intermolecular interactions contribute to the G4-like spectral features observed for T4 at high concentration. To examine whether T4 also shows a tendency toward higher order assembly after preparation at high concentration, morphological observations by atomic force microscopy (AFM) were performed, as shown in Figure S4. Topographic images of TBA at 0.1 mg/mL show numerous isolated spherical spots, most of which have a maximum height of less than 2 nm. Their morphology and height are consistent with intramolecular G4 structures.^46^^,^^47^^,^^48^^,^^49^ Conversely, the AFM topography for T4 samples initially prepared at 50 mg/mL is markedly different from that for TBA. Instead of discrete dots, large aggregates with a characteristic height of approximately 8 nm, exceeding the height expected for single G-wires,^49^^,^^50^ are clearly resolved. These aggregates extend into elongated, linear structures that form intricate, fiber-like, interconnected networks. These observations are consistent with the presence of thicker bundles or multi-stranded fiber networks in the dried specimens. In contrast, no distinct nanostructures are observed by AFM for T4 at 0.1 mg/mL (data not shown). Because the samples were substantially diluted, deposited onto mica, washed, and dried before AFM imaging, the observed aggregates and fiber-like structures should not be interpreted as a direct representation of the assembly state in the original 50 mg/mL solution. Furthermore, AFM does not provide information on molecular stoichiometry or establish that the observed assemblies represent intermolecular G4 structures. Despite these limitations, the AFM observations support an increased tendency of T4 to form higher order assemblies after incubation at high concentration. Together with the G4-like spectroscopic features observed at high concentration, these findings suggest that the concentration-dependent structural changes in T4 may be associated with higher order assembly. Thus, sequences with low apparent G4-forming propensity at low concentrations may still adopt G4-like or higher order G-rich structures under highly concentrated conditions.

This observation has important implications for the formulation of antisense oligonucleotides for which HOS formation is undesirable, and it indicates that even sequences that appear unlikely to form such structures should be examined at formulation-relevant concentrations to ensure that they do not form.

To objectively evaluate the concentration-dependent changes observed in the CD spectra, a principal component analysis (PCA) classification was performed (Figure 2). The classification model was created by applying PCA to 30 G4 CD spectra with identified structures and performing hierarchical clustering of the resulting PCA scores (Figure S5). As shown in Figure 2A, the results for TBA remain within the 95% probability region constructed from the antiparallel reference spectra at all concentrations, indicating no substantial topological changes. The PCA scores for T1 fall outside the 95% probability region at 0.1 mg/mL but shift into the region at 5 mg/mL (Figure 2B). These spectral changes indicate a concentration-dependent shift toward a more parallel-like CD spectral profile. The PCA scores for T2 and T3 are located within the antiparallel 95% probability region at 0.1 mg/mL; however, they shift toward the parallel G4 cluster as the concentration increases (Figures 2C and 2D). At 50 mg/mL, both T2 and T3 further shift toward the parallel region, with T3 positioned closer to the cluster than T2. The PCA score for T4 falls within the parallel 95% probability region at 50 mg/mL (Figure 2E).

**Figure 2:**
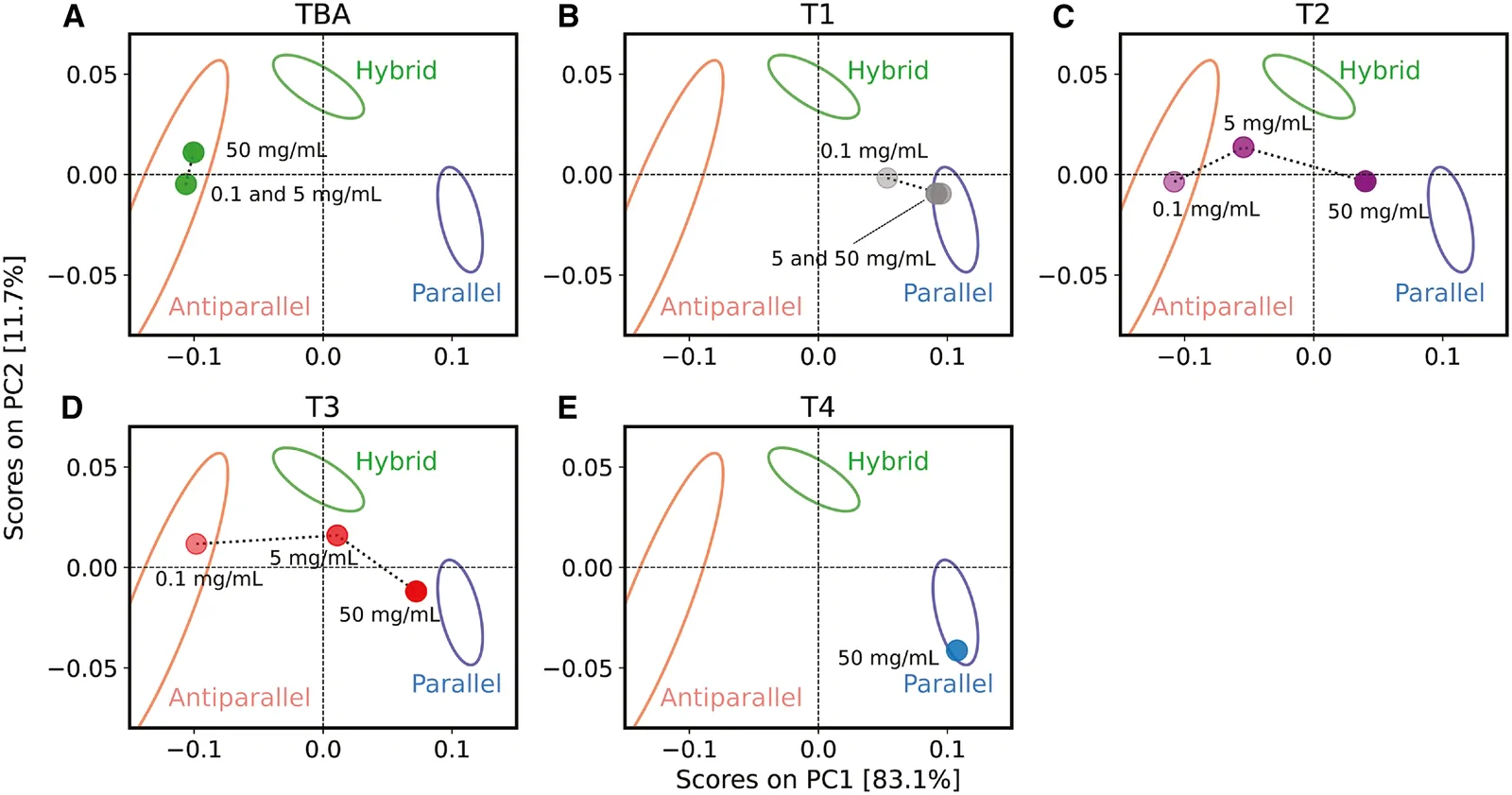
PCA score plots based on CD spectra of TBA and modified TBAs (A) TBA, (B) T1, (C) T2, (D) T3, and (E) T4. Each cluster was defined based on hierarchical clustering of the reference spectra, and the 95% probability ellipses are shown. Each PCA score represents the mean of three measurements.

### Raman spectra of nucleic acids

In the Raman spectra, distinct vibrational bands in the fingerprint region (1,800–600 cm^−1^) are clearly observed at concentrations of both 5 and 50 mg/mL (Figure 3). In all TBA and modified TBA samples, a characteristic band is consistently detected near 1,480 cm^−1^ (Figure 4A). This band is attributed to dG-N7 involved in Hoogsteen hydrogen bonding.^21^^,^^51^^,^^52^ It has been reported that this band appears several cm^−1^ lower in wavenumber than that arising from weak hydrogen bonding between dG-N7 and water molecules.^21^ Only minor wavenumber shifts are observed when the concentration is increased from 5 to 50 mg/mL for TBA, T1, T2, and T3, with shifts of −0.6, 0.0, −0.7, and −0.4 cm^−1^, respectively, and statistical testing does not detect significant differences in these band positions. Notably, T4 shows a pronounced shift of −1.9 cm^−1^ at the higher concentration of 50 mg/mL, which is statistically significant (*p* < 0.05) (Figure 4B). This substantially larger shift compared with those for TBA and the modified TBA sequences is likely associated with a change in the hydrogen-bonding environment of dG-N7, from weak hydrogen bonding with water molecules to Hoogsteen hydrogen bonding.^21^ Such a shift suggests that T4 undergoes a more pronounced conformational rearrangement than the other oligonucleotides, potentially involving a transition from an unidentified non-G4 structure to a G4-like conformation. This interpretation is consistent with the CD results, which show a transition toward a parallel-type G4-like spectrum upon increasing the concentration from 5 to 50 mg/mL (Figure 1E).

**Figure 3:**
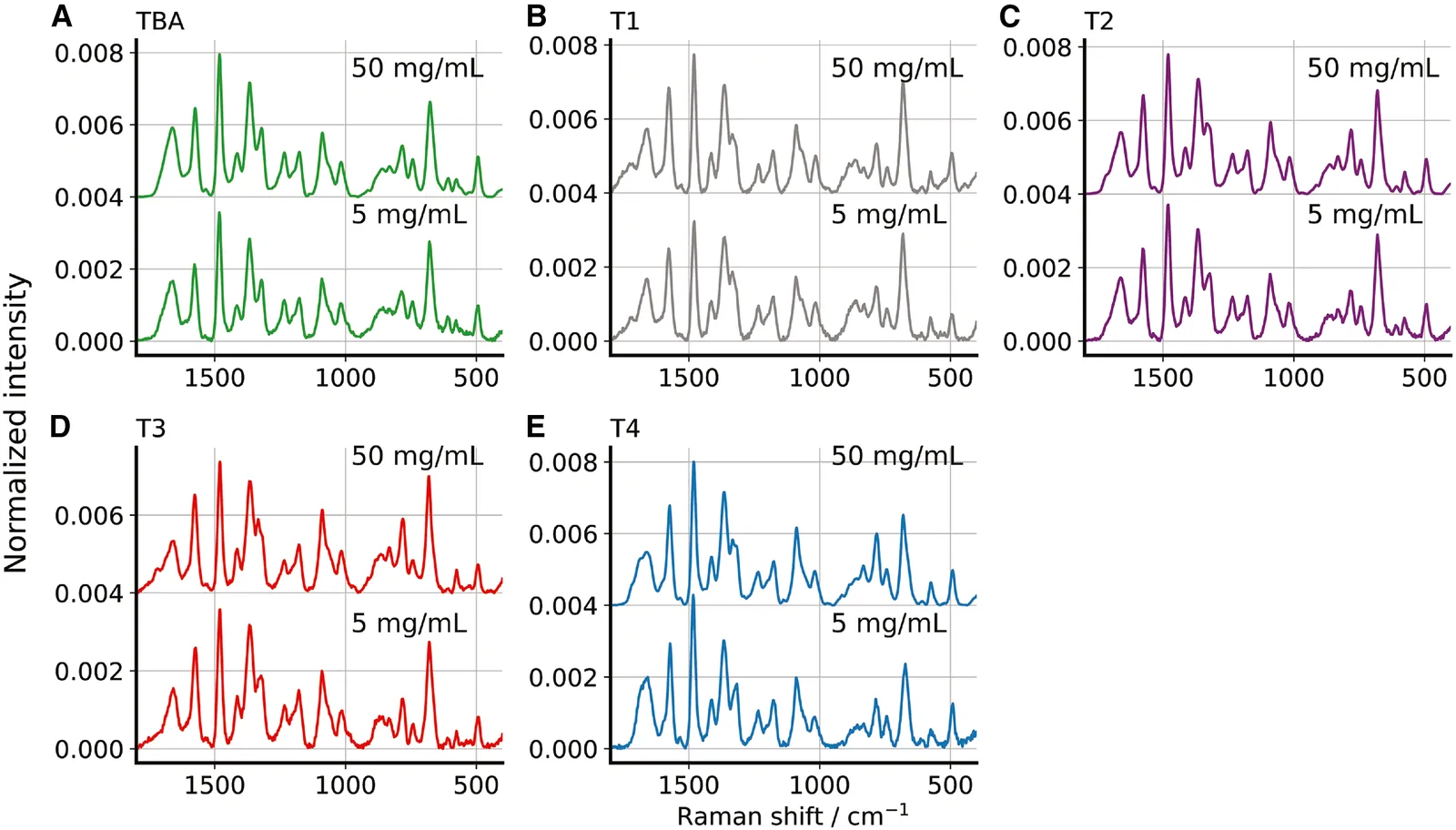
Raman spectra of TBA and modified TBAs (A) TBA, (B) T1, (C) T2, (D) T3, and (E) T4. Spectra recorded at 5 mg/mL (lower) and 50 mg/mL (upper) are shown with vertical offsets for clarity. Each spectrum represents the mean of three measurements.

**Figure 4:**
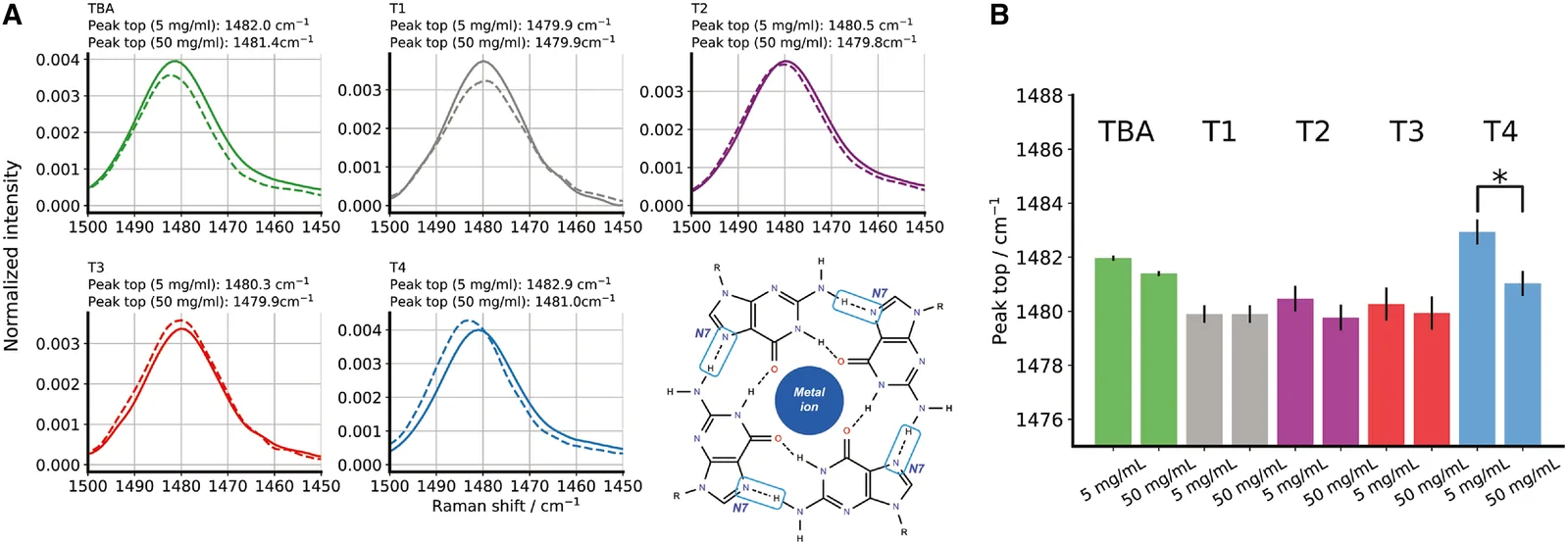
Raman spectra of TBA and modified TBAs in the vicinity of 1,480 cm^−1^ (A) Raman spectra of TBA and modified TBAs at 5 and 50 mg/mL, and the structure of the G-quartet with the dG-N7 atoms involved in Hoogsteen hydrogen bonding highlighted in cyan. Each spectrum represents the mean of three measurements. Spectra recorded at 5 and 50 mg/mL are shown as dashed and solid lines, respectively. (B) Peak top positions and SDs calculated from three measurements. Asterisks indicate statistically significant differences (*p* < 0.05).

The Raman bands near 680 cm^−1^, which are associated with the base orientation, were compared to evaluate topology changes in TBA and the modified TBA sequences (Figure S6).^21^^,^^51^^,^^52^ The band tops for T1, T2, and T3 are located at higher wavenumbers than that for TBA. It has been reported that bases adopting an anti-orientation, characteristic of parallel G4, give rise to bands at higher wavenumbers than syn-oriented bases that are partially present in antiparallel structures.^21^^,^^51^^,^^52^ The observed band tops are consistent with the CD results, which indicate that TBA forms an antiparallel topology, whereas T1 and T3 are dominated by a parallel-like topology and T2 includes a partial parallel-like contribution within this concentration range.

In contrast, T4 shows a Raman band at 672.4 cm^−1^ at 5 mg/mL, which is markedly different from those for TBA and the modified TBA sequences. Upon increasing the concentration to 50 mg/mL, the band shifts significantly to a higher wavenumber of 680.0 cm^−1^ (*p* < 0.05), coinciding with the band positions for T1, T2, and T3. These results suggest that T4 may adopt a more parallel G4-like or anti-oriented base arrangement at 50 mg/mL than at 5 mg/mL.

Raman bands near 1,330 cm^−1^, which are highly sensitive to the base orientation, were also compared (Figure S7). A distinct band near 1,335 cm^−1^, attributed to bases adopting an anti-orientation,^21^^,^^51^^,^^52^ is not evident for TBA, whereas this band is clearly detected for T1, T2, and T3. In addition, the intensity of this band increases for T2, T3, and T4 when the concentration is increased from 5 to 50 mg/mL.

### ATR FT-IR spectra of nucleic acids

As shown in Figure 5, the ATR FT-IR spectra of TBA and modified TBAs measured at 50 mg/mL exhibit well-defined bands originating from nucleobases in the 1,800–1,600 cm^−1^ region and from the phosphate backbone and deoxyribose near 1,100 cm^−1^.^33^^,^^53^

**Figure 5:**
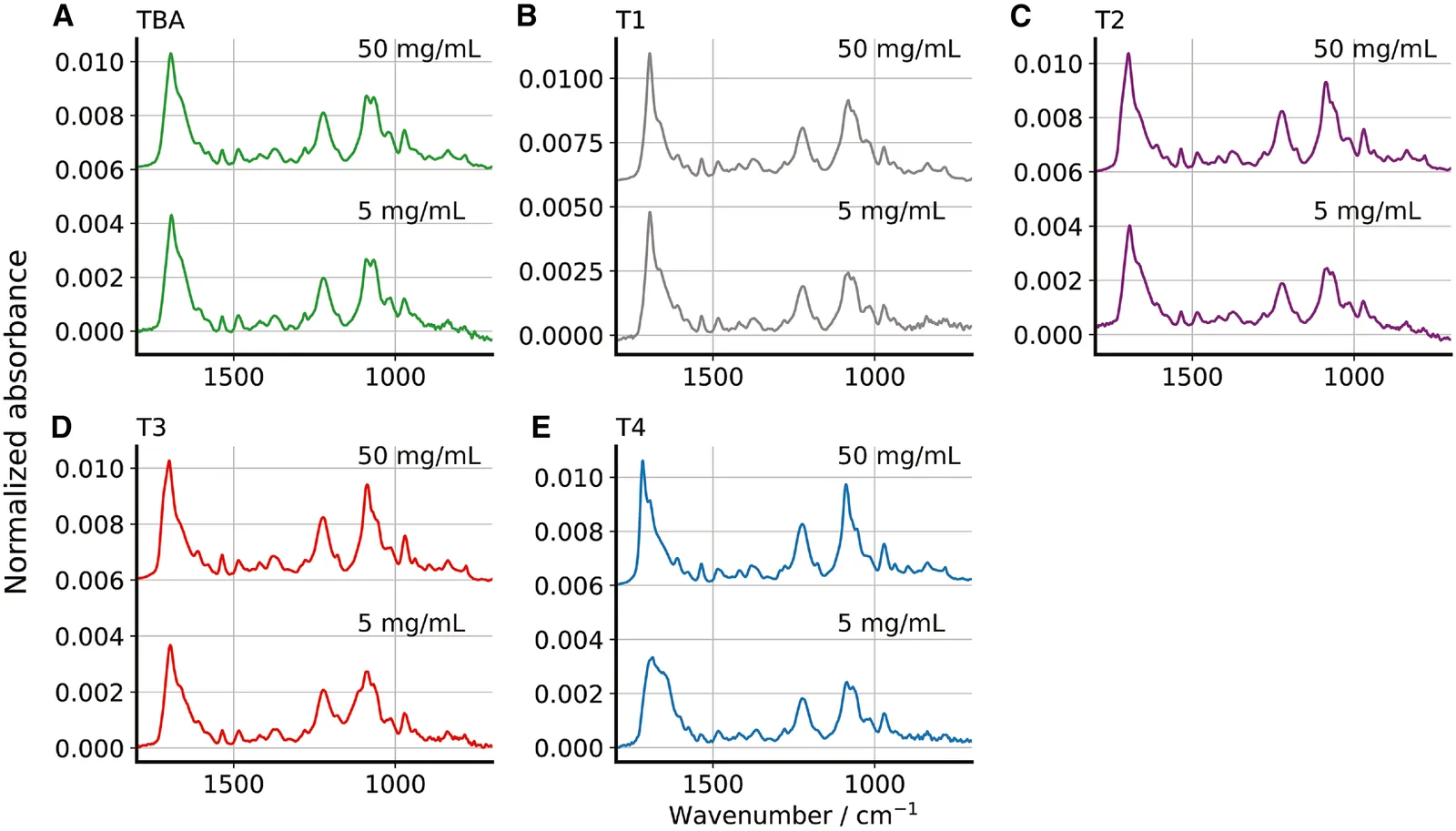
ATR FT-IR spectra of TBA and modified TBAs (A) TBA, (B) T1, (C) T2, (D) T3, and (E) T4. Spectra recorded at 5 mg/mL (lower) and 50 mg/mL (upper) are shown with vertical offsets for clarity. Spectra for 5 and 50 mg/mL represent the mean of five and three measurements, respectively.

At 50 mg/mL, a band near 1,537 cm^−1^ is clearly observed for TBA and all modified TBAs (Figure 6, purple area). This band is assigned to C=N and C–N stretching vibrations, including contributions from N7 involved in Hoogsteen hydrogen bonding, which has been reported as a spectral feature associated with G4 formation.^53^ At 5 mg/mL, the corresponding band is observed for TBA, T1, T2, and T3, whereas the band for T4 is less clearly resolved (Figure 6B, purple area). Similarly, a band at 1,690 cm^−1^, assigned to C6=O6 carbonyl stretching associated with Hoogsteen dG interactions,^33^ is observed for TBA and all modified TBAs at 50 mg/mL (Figure 6A, orange area). In contrast, at 5 mg/mL, the corresponding band for T4 is broad and less distinct (Figure 6B, orange area). In addition, at 50 mg/mL, an unassigned feature near 1,720 cm^−1^ is found, whose intensity differs among TBA and the modified TBAs (Figure 6A, green area). TBA shows a slight shoulder, T2 and T3 show clear shoulder features, and T4 shows a pronounced band.

**Figure 6:**
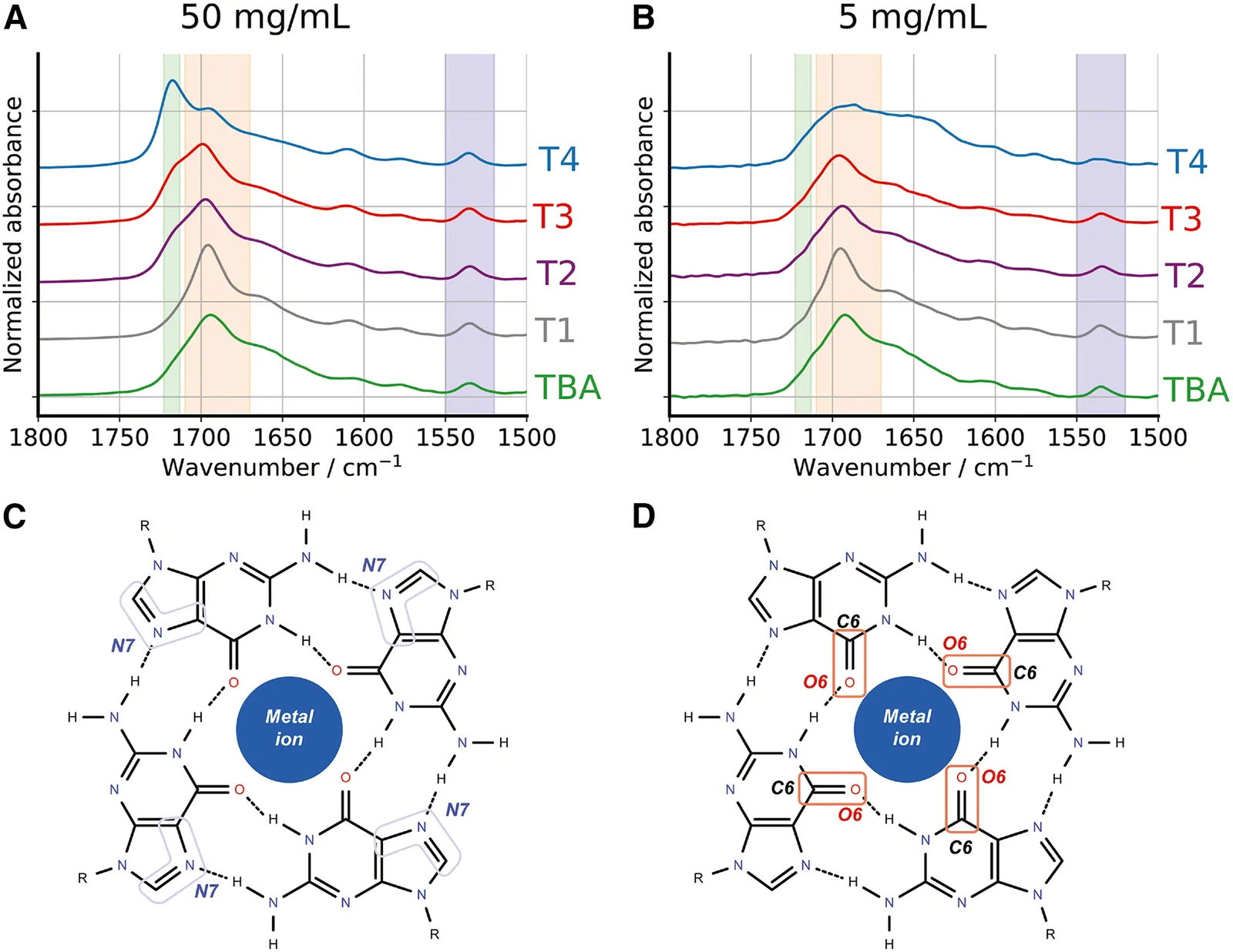
ATR FT-IR spectra of TBA and modified TBAs in the vicinity of 1,700 cm^−1^ (A and B) Spectra at (A) 50 mg/mL and (B) 5 mg/mL. (C and D) Structure of the G-quartet. Regions assigned to C=N and C–N stretching vibrations and to C6=O6 stretching vibrations are highlighted in purple and orange, respectively, both in the spectra and in the structure of the G-quartet. The band at 1,720 cm^−1^, whose assignment could not be determined, is highlighted in green. Spectra for 5 and 50 mg/mL represent the means of five and three measurements, respectively.

In Figure S8, in the region around 1,100 cm^−1^, two overlapping bands are observed: one near 1,090 cm^−1^, attributed to tetraplex formation, and another near 1,077 cm^−1^, attributed to loop regions or non-base-paired structures.^53^ For TBA, the ratio of the intensity of the bands at approximately 1,090 and 1,070 cm^−1^ is 1.03 at 5 mg/mL and 1.04 at 50 mg/mL, and no statistically significant difference in this ratio is detected between the two concentrations (*p* > 0.05), indicating no apparent concentration dependence. A similar trend is observed for T1, with intensity ratios of 1.06 at 5 mg/mL and 1.04 at 50 mg/mL; no statistically significant change in this ratio is detected between the two concentrations (*p* > 0.05). In contrast, the intensity ratio changes significantly with concentration for both T2 and T3 (*p* < 0.05 for each). The ratio for T2 increases from 1.06 at 5 mg/mL to 1.26 at 50 mg/mL, while that for T3 increases from 1.20 to 1.36 over the same concentration range. These results are consistent with the CD data, which show no apparent concentration-dependent change in the topology of TBA, only minor changes for T1, and concentration-dependent shifts toward parallel-type topologies for T2 and T3. For T3 at a concentration of 5 mg/mL, an additional shoulder with no clear assignment appears in the IR spectrum near 1,120 cm^−1^, but this band is no longer seen at a concentration of 50 mg/mL.

T4 exhibits a particularly pronounced and statistically significant change in band intensity ratio (*p* < 0.05), with values of 1.06 at 5 mg/mL and 1.53 at 50 mg/mL. This result is consistent with the changes in the CD spectra and the distinct band shifts detected by Raman spectroscopy.

### Structure reversibility

Therapeutic oligonucleotides are typically administered as high-concentration formulations but are rapidly diluted *in vivo* after administration. To examine dilution-induced structural changes, T1, T2, T3, and T4 were selected because they exhibited concentration-dependent structural changes. Each modified TBA was diluted from 50 to 0.1 mg/mL, and time-dependent CD spectra were measured immediately after dilution. Figure 7 shows representative time-dependent changes in the CD intensity at 262 nm, with the full time-dependent CD spectra shown in Figures S9 and S10.

**Figure 7:**
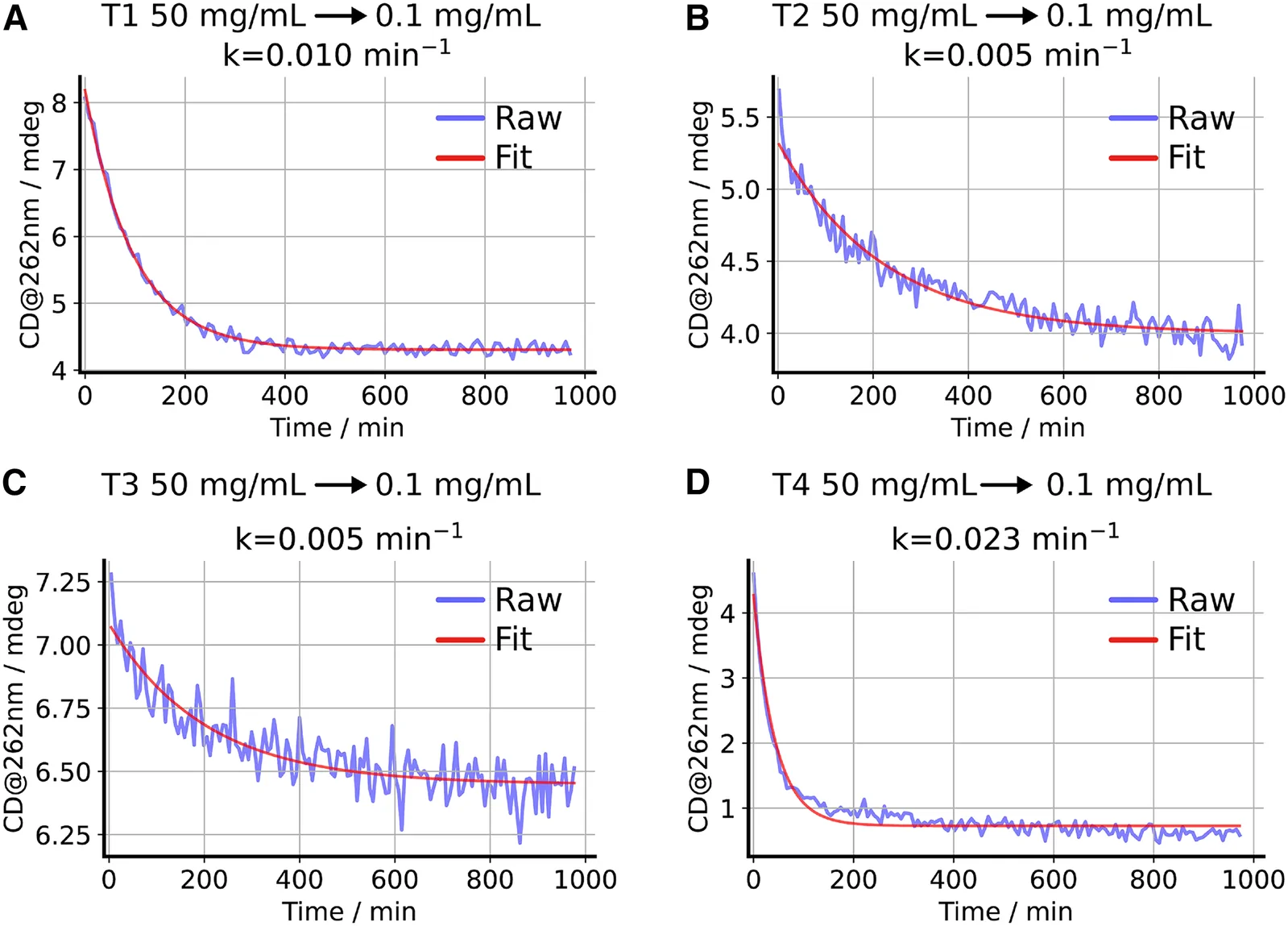
Time-dependent structural changes upon dilution Time-dependent changes in CD intensity at 262 nm after dilution from 50 to 0.1 mg/mL for (A) T1, (B) T2, (C) T3, and (D) T4. Blue lines indicate the experimental CD values at 262 nm, and red lines indicate the fitting curves obtained using a first-order kinetic model.

Notably, the dilution-induced structural changes differ among the modified TBAs. The CD spectra of T1 and T4 eventually become comparable to those observed at 0.1 mg/mL (Figures S9A and S10B), whereas T2 and T3 show structural changes after dilution, but their spectra do not return to those at 0.1 mg/mL (Figures S9B and S10A). For T1 and T4, the time-dependent changes in characteristic CD intensity after dilution from 50 to 0.1 mg/mL give apparent rate constants of 0.010 and 0.023 min^−1^, respectively (Figures 7A and 7D). These results indicate that the structures change on a timescale of tens of minutes and that the rate of structural change differs by more than 2-fold depending on the modified TBA.

## Discussion

This study suggests that CD spectroscopy can provide a practical approach for evaluating nucleic acid HOS across a range of concentrations. In addition, the application of PCA combined with clustering enables detailed and objective discrimination of G4 topologies. Nevertheless, the observation of unexpected HOS formation, as exemplified by T4, highlights the importance of validating CD-based interpretations using orthogonal vibrational spectroscopic techniques such as Raman and ATR FT-IR spectroscopy. These complementary methods probe structural changes through nucleobase and phosphate backbone vibrations, thereby increasing confidence in the structural assignments derived from CD measurements.

In the ATR FT-IR spectra of TBA, T2, and T3, a band at 1,720 cm^−1^, located at a slightly higher wavenumber than the C6=O6 carbonyl stretching band at 1,690 cm^−1^, was observed as a shoulder, whereas it appeared as a distinct band for T4 (Figure 6). Although a definitive assignment of this band could not be made, its distinct appearance in the T4 spectrum suggests that the hydrogen-bonding environment for the O6 carbonyl group and N1 may differ in T4 compared with TBA and the modified TBAs.

In addition, T3 exhibited a pronounced band at a higher wavenumber than the phosphate backbone-associated band near 1,090 cm^−1^ (Figure S8). Because this feature could not be readily assigned to known vibrational modes, it may reflect differences in the local environment or conformational state of the phosphate backbone in T3 relative to the other sequences.

While Raman and ATR FT-IR spectroscopy inherently involve some subjectivity in band assignment, the establishment of reference-based classification frameworks analogous to those used in CD spectroscopy would enable more objective and pharmaceutical-relevant evaluation of nucleic acid HOS. Databases for nucleic acid structures have already been reported for infrared spectroscopy,^35^ and the development of comparable, standardized reference datasets for Raman spectroscopy would further support orthogonal assessment strategies for the characterization of nucleic acid therapeutics.

Using a cell with an optical path length of 0.01 mm, CD measurements of oligonucleotide samples at concentrations up to 50 mg/mL were feasible. The CD and high-tension (HT) voltage spectra of DPBS measured using each cell are shown in Figure S13. However, when measuring such highly concentrated samples, the viscosity and refractive index of the samples may cause distortion of the CD spectra and flattening of the absorption spectra. To assess possible spectral artifacts under highly concentrated conditions, IgG was used as a model sample whose HOS was not expected to change substantially over the tested concentration range. Near-UV CD spectra of IgG were measured from 5.8 to 156.2 mg/mL, covering a concentration range higher than that used for the TBA measurements. No apparent distortion was observed in either the CD spectra or HT voltage spectra under these conditions (Figures S14A and S14B). However, in one measurement of T3 at 50 mg/mL, high viscosity was accompanied by spectral distortion and absorbance flattening (Figures S14C and S14D). This phenomenon was not reproducibly observed in every measurement but occurred occasionally, and its underlying cause remains unclear. Throughout the analysis, the CD spectra, HT voltage spectra, and absorption spectra were carefully inspected to identify possible artifacts, and spectra showing such distortions were excluded from the analysis. On the other hand, these changes in sample properties themselves may affect formulation studies. Therefore, elucidating the origin of these occasionally observed phenomena would be useful for future formulation studies. Collectively, these results suggest that at oligonucleotide concentrations above 50 mg/mL, although Raman and ATR FT-IR spectroscopy may provide more robust structural evaluation, the development of CD measurement approaches compatible with shorter optical path lengths is desirable.

To characterize the HOS under high-concentration formulation conditions, the amount of sample required for measurement becomes a critical factor. At a concentration of 50 mg/mL, the measurement volumes used in this study were 7 μL for CD, 5 μL for FT-IR, and 7 μL for Raman spectroscopy.

For CD spectroscopy, further reduction of the sample volume would require the development of cells that can achieve ultra-short path lengths while incorporating small apertures, as implemented in microcells such as the MSD-462 cell (JASCO Corporation) and the DMV Bio-cell (Starna Scientific, Ltd.).^38^^,^^54^ In addition, focusing the excitation beam using optical lenses would allow the sample volume to be reduced while maintaining an adequate signal-to-noise ratio in the measured spectra.^54^ In the case of FT-IR spectroscopy, measurements were performed in an open system using the ATR method. Therefore, reducing the sample volume raises concerns regarding solvent evaporation. The introduction of a sealed measurement mechanism that suppresses evaporation would potentially allow measurements at volumes lower than 5 μL. Raman spectroscopy, by contrast, is inherently well suited to low-volume measurements because it is based on a microscopic optical system. The use of high-magnification objective lenses, combined with a reduced sample plate volume, would readily enable further reductions in sample volume. Consequently, among the three techniques evaluated, Raman spectroscopy appears to offer the greatest practical feasibility for additional sample volume minimization. Moreover, the establishment of Raman spectral libraries, similar to those established for CD, would enable objective HOS assessment and is expected to provide substantial benefits for formulation studies of nucleic acid therapeutics.

The observation that T4 showed G4-like spectral features at high concentration highlights the potential value of evaluating concentration-dependent structural behavior during oligonucleotide formulation development. For antisense therapeutics, in which unintended HOS formation may negatively affect potency, stability, or manufacturability, assessing structural behavior at formulation-relevant concentrations should be considered during formulation development.

Because TBA is an aptamer, the present findings cannot be directly generalized to antisense oligonucleotides and siRNA, which currently represent a major class of approved nucleic acid therapeutics. Nevertheless, it is notable that T4, which was designed to avoid G4 formation by limiting consecutive guanine residues to no more than two, exhibited concentration-dependent G4-like spectroscopic features. Although AFM did not directly image T4 in its original high-concentration solution state, higher order assemblies were observed after incubation at high concentration, whereas no distinct nanostructures were observed at low concentration, supporting a concentration-dependent tendency of T4 to form higher order assemblies. Taken together, these results suggest that the concentration-dependent G4-like structural changes in T4 are associated with higher order assembly. However, the present data cannot conclusively establish whether these assemblies represent intermolecular G4 structures. Nevertheless, these findings suggest that evaluating HOS under formulation-relevant conditions may also be useful for antisense oligonucleotides containing consecutive guanine residues.

Among the HOSs formed by the modified TBAs at 50 mg/mL, those of T2 and T3 showed only limited structural changes after dilution and persisted for at least 900 min. Although T1 and T4 underwent dilution-induced structural transitions, these changes occurred on a minute-to-hour timescale rather than immediately. These findings suggest that the concentration-dependent HOS formed in therapeutic oligonucleotide formulations may be partially retained after dilution upon administration. Therefore, these results suggest the potential usefulness of evaluating HOS formation under formulation-relevant concentration conditions.

In addition, the concentration-dependent structural changes observed between 0.1 and 5 mg/mL have important implications for NMR-based structural characterization of nucleic acids. Because NMR experiments are typically conducted at concentrations above approximately 200 μM (≈1 mg/mL), differences in sample concentration between NMR and other analytical assays may lead to the observation of distinct HOSs. In this context, the use of CD spectroscopy to systematically assess concentration-dependent structural transitions, as demonstrated in this study, provides a valuable framework for correlating structure with biological activity and analytical readouts in nucleic acid therapeutics.

CD spectroscopy provided an efficient and objective means to classify G4 topologies using established reference-based models, while Raman and ATR FT-IR spectroscopy offered complementary insights into nucleobase orientation, hydrogen-bonding environments, and phosphate backbone conformations. Importantly, the combined use of these orthogonal techniques enabled the validation of CD-based structural interpretations and revealed subtle but significant structural transitions that would not be apparent using a single analytical method alone.

These findings highlight the necessity of evaluating nucleic acid HOS under concentration conditions relevant to pharmaceutical formulation. The orthogonal spectroscopic framework presented here provides a practical and broadly applicable platform for screening concentration-dependent structural behavior and has direct implications for the development, quality assessment, and risk mitigation of nucleic acid therapeutics.

## Materials and methods

### Sample preparation

TBA and modified TBAs were designed to control intra-/intermolecular interactions based on the G4 structure (Table 1) and were synthesized by Hokkaido System Science (Sapporo, Japan). All oligonucleotides used in this study were high-performance liquid chromatography-purified grade, with their counter cations exchanged from triethylamine to sodium. Each oligonucleotide was diluted using Dulbecco’s phosphate-buffered saline (DPBS; 8.1 mM Na_2_HPO_4_, 2.68 mM KCl, 1.47 mM KH_2_PO_4_, and 137 mM NaCl) purchased from Nacalai Tesque (Kyoto, Japan). The concentrations of the oligonucleotides were determined by NanoDrop spectrophotometric measurement at 260 nm. Each TBA and modified TBA was heat treated at 95°C for 5 min and then gradually cooled at a rate of 1°C min^−1^ to 4°C using a thermal cycler (PCR Thermal Cycler Dice Standard TP650, Takara, Kyoto, Japan) to form G4 structures. The G4-folded oligonucleotides were stored at 4°C until further use.

### CD spectroscopy

CD spectra of TBA and modified TBAs were acquired using a J-1500 CD spectrometer (JASCO Corporation, Tokyo, Japan) with a 1-mm cell and a PTC-510 Peltier cell holder (JASCO Corporation) for 0.1 mg/mL oligonucleotides, a 124-QS 0.1-mm demountable cell (Hellma GmbH & Co. KG, Jena, Germany) and standard cell holder for 5 mg/mL oligonucleotides, and a 124-QS 0.01-mm demountable cell (Hellma GmbH & Co. KG) and standard cell holder for 50 mg/mL oligonucleotides, at room temperature. The wavelength range was 220–320 nm, the bandwidth was 1 nm, the data integration time was 4 s, and the scan speed was 50 nm/min with four accumulations. Spectra were baseline corrected by subtracting the spectrum of DPBS recorded under identical conditions, offset corrected so that the CD value at 320 nm became zero, and normalized by the area of the spectrum. The spectral area was calculated as the sum of the absolute values of the spectral intensities over the measured wavelength range. Measurements were performed three times for each oligonucleotide.

#### Raman spectroscopy

Raman spectra of TBA and modified TBAs were acquired using an NRS-4500 (JASCO Corporation) with a custom-designed sample plate (Figure S11) at room temperature. The samples were measured at concentrations of 5 and 50 mg/mL. Oligonucleotide solutions were dropped onto the plate and covered by an 18 × 18 mm cover glass (Matsunami, Osaka, Japan). Before measurement, the wavenumber was calibrated using the 520 cm^−1^ band for silicon. Oligonucleotides were excited using a 532 nm laser at 40 or 80 mW, with the laser power adjusted to prevent saturation of the charge-coupled device (CCD), through a 50× objective lens. The signal was detected using a 900 grooves/mm grating and a 1,024-channel CCD. The exposure time was 10 s, and the number of accumulations was 64. Spectra were baseline corrected by subtracting the spectrum of DPBS recorded under identical conditions and were fluorescence corrected using the fluorescence correction function of Spectra Manager version 2.5 (JASCO Corporation). The spectra were normalized by their area, and a Savitzky-Golay filter (window length: 15 cm^−1^, polynomial order: 2) was applied. Measurements were performed three times for each oligonucleotide.

#### FT-IR spectroscopy

ATR FT-IR spectra of TBA and modified TBAs were acquired using an FT/IR-6X equipped with the ATR PRO ONE ATR measurement unit (JASCO Corporation) at room temperature. The samples were measured at concentrations of 5 and 50 mg/mL. The ATR prism was a single-reflection diamond crystal. The instrument was purged with N_2_ during measurements. Spectra were recorded at a resolution of 4 cm^−1^ with 128 scans. Spectra were baseline corrected by subtracting the spectrum of DPBS recorded under identical conditions. ATR FT-IR spectra were corrected for water vapor and anomalous dispersion using Spectra Manager 2.5 (JASCO Corporation) and normalized by their area. For each oligonucleotide, measurements were performed five times at 5 mg/mL and three times at 50 mg/mL.

#### ThT assay

Oligonucleotide samples at 0.1 or 50 mg/mL in DPBS (10 μL) were mixed with 1 μL of 40 μM ThT (Sigma-Aldrich). After incubation for 5 min at room temperature, fluorescence spectra were recorded using an FP-8350 spectrofluorometer equipped with an SAF-851 one-drop measurement unit (JASCO Corporation; Figure S12). The excitation wavelength was 440 nm, and emission spectra were collected from 460 to 530 nm at a scan speed of 50 nm/min. The excitation and emission bandwidths were both set to 5 nm, and the number of accumulations was set to three. Measurements were performed three times for each oligonucleotide.

### PCA of CD spectra

CD spectra of 22 reference G4 reported by Del Villar-Guerra et al. were combined with additional eight G4 spectra,^28^ and detailed information on the reference G4 structures is summarized in Table S1.

CD spectra of TBA and its variants were acquired using a demountable cell. Because the optical path length can vary between measurements due to cell assembly, the acquired CD spectra were normalized by the area of the absolute spectral intensity to minimize path-length-related errors. Accordingly, the reference spectra used for PCA were also normalized in the same manner as a preprocessing step. The normalized reference spectra were then mean centered, and PCA scores were calculated as the inner products between the mean-centered normalized spectra and the corresponding eigenvectors.

Del Villar-Guerra et al. reported that five principal components are sufficient to describe the reference G4 CD spectra.^28^ In the present study, we added our own reference spectra to the dataset and applied the same preprocessing procedure. We confirmed that the cumulative explained variance exceeded 99.9% when five principal components were used, indicating that these five components could explain more than 99.9% of the variance in this dataset. Therefore, the scores for PC1 to PC5 were used for hierarchical clustering of the reference spectra using Euclidean distance as the distance metric and Ward’s method as the linkage algorithm. For visualization of the score distributions in the PC1-PC2 plane, PC1 and PC2 scores for each cluster were plotted with 95% probability ellipses, assuming a bivariate normal distribution. The ellipses were calculated from the covariance matrix of the PC1 and PC2 scores, with the scaling factor determined from the 95th percentile of the chi distribution with two degrees of freedom. CD spectra of reference and PCA scores are shown in Figure S5. All analyses were conducted using scikit-learn version 0.24.2.^55^

#### Statistical testing

Statistical analyses were performed to evaluate differences in Raman peak positions and FT-IR peak positions and peak ratios between concentrations. Two-sided Welch’s *t* tests were used for all comparisons because equal population variances between the compared groups were not assumed. No correction for multiple comparisons was applied. Normality was assessed using the Shapiro-Wilk test for all datasets used for the statistical comparisons (30 datasets in total). Five of the 30 datasets showed *p* values below 0.05, whereas no significant deviation from normality was detected for the remaining 25 datasets. Considering that most datasets showed no significant deviation from normality and that the small sample sizes limited rigorous assessment of the underlying distributions, Welch’s *t* test was applied consistently to all comparisons. All statistical analyses were performed using SciPy version 1.16.0.^56^

The replicate design for the statistical comparisons was as follows. For FT-IR measurements, five samples were analyzed at 5 mg/mL (*n* = 5) and three samples at 50 mg/mL (*n* = 3). Among the five samples at 5 mg/mL, two samples were prepared on one day and the remaining three on another day. Among the three samples at 50 mg/mL, one sample was prepared on one day and the remaining two on another day. For Raman measurements, three samples were analyzed at each concentration (*n* = 3); at both 5 and 50 mg/mL, one sample was prepared on one day and the remaining two on another day.

#### AFM

Topographic measurements were performed using a JPK NanoWizard 4XP AFM (Bruker, Billerica, MA, USA). AFM images were obtained in the Quantitative Imaging (QI) mode using ScanAsyst-Air cantilevers (Bruker). The nominal spring constant and resonance frequency of the cantilever were 0.4 N/m and 70 kHz, respectively, whereas the actual calibrated spring constant was 288 mN/m and the resonance frequency was approximately 72 kHz. Images were acquired with a scan size of 600 × 600 nm at a resolution of 256 × 256 pixels using a setpoint of 5.0 nN. Image analysis was performed using the JPK Data Processing software (Bruker).

To enhance aptamer-mica binding, the mica substrates were pre-incubated with 5 mM NiCl_2_ for 2 min, rinsed with Milli-Q water, and dried using a gentle stream of nitrogen gas. Refolded TBA at 0.1 mg/mL or T4 at 50 mg/mL was diluted to a concentration of 1 ng/μL in DPBS. Subsequently, 10 μL of the diluted oligonucleotide solution was deposited onto the pre-treated mica surface, incubated for 5 min at room temperature, washed with Milli-Q water, and dried under vacuum.

### Time-course CD measurements after dilution

T1, T2, T3, and T4 solutions at 50 mg/mL were diluted to 0.1 mg/mL with DPBS and promptly subjected to time-course CD measurements using a J-1500 CD spectrometer equipped with a PTC-510 Peltier cell holder or an MPTC-513 multi-position Peltier cell changer (JASCO Corporation) at room temperature. CD spectra were continuously recorded for more than 900 min using a 1 mm path-length cell over the wavelength range of 220–320 nm. The bandwidth was set to 1 nm, the integration time was 4 s, the scan speed was 50 nm/min, and the number of accumulations was set to one.

## Data and code availability

The raw data are available through the National Institute for Materials Science Materials Data Repository: https://doi.org/10.48505/nims.6486.

## Acknowledgments

We thank Ms. Tomoyo Umezawa and Dr. Miwako Shobo (National Institute for Materials Science [NIMS]) for their excellent technical assistance. Part of this work was conducted at the NIMS Molecule and Material Synthesis Platform, supported by the Nanotechnology Platform Program of the 10.13039/501100001700Ministry of Education, Culture, Sports, Science and Technology, Japan. This work was partly funded by the Materials Open Platform for Pharmaceutical Science at the 10.13039/501100004496National Institute for Materials Science and also by the 10.13039/501100001691Japan Society for the Promotion of Science 10.13039/501100001691KAKENHI, grant no. 23K18527.

## Author contributions

T.O., methodology, formal analysis, visualization, writing – original draft, and writing – review and editing; T.Y., conceptualization, visualization, writing – original draft, writing – review and editing, and supervision; S.S., validation and formal analysis; K.-i.A., investigation and supervision; K.I., supervision, project administration, and writing – review and editing; K.K., conceptualization, writing – review and editing, project administration, and funding acquisition.

## Declaration of interests

T.O., S.S., and K.-i.A. are employees of JASCO Corporation.

## Supplemental information

Document S1. Figures S1–S14 and Table S1

Document S2. Article plus supplemental information
