# Supplementary material for: Orthogonal spectroscopic assessment of higher order structure of nucleic acids and its concentration-dependent polymorphism
Source: Mol Ther Nucleic Acids. 2026 Oct 7;37(4):103131. doi: 10.1016/j.omtn.2026.103131 (PMC13756148; doi:10.1016/j.omtn.2026.103131)
Supplement: Supplementary file 1 [file mmc1.pdf]

OMTN, Volume *37*

Supplemental information

Orthogonal spectroscopic assessment of higher order structure of nucleic acids and its concentration-dependent polymorphism

Taiji Oyama, Satoko Suzuki, Ken-ichi Akao, Kazunori Ikebukuro, Kohsaku Kawakami, and Tomohiko Yamazaki

Supplemental information

Table S1. Information on the reference nucleic acid CD spectra used to construct the PCA model. The spectra were kindly provided by Drs. Trent and Chaires. Asterisks indicate reference nucleic acid CD spectra independently added in this study.

PDB ID	Sequence (5'->3')	Method	Topology
1EMQ	TGGTGGC	NMR	parallel
1EVM	AGGGT	NMR	parallel
1FQP	GGGTTTTGGG	NMR	antiparallel
1I34	GGTTTTGGCAGGGTTTTGGT	NMR	antiparallel
1LVS	GGGGTTTTGGG	NMR	antiparallel
1NP9	TTAGGGT	NMR	parallel
1XAV	TGAGGGTGGGTAGGGTGGGTAA	NMR	parallel
139D	TTGGGGT	NMR	parallel
148D	GGTTGGTGTGGTTGG	NMR	antiparallel
201D	GGGGTTTTGGGGTTTTGGGGTTTTGGGG	NMR	antiparallel
156D	GGGGTTTTGGGG	NMR	antiparallel
1S45	TGGGGT	X-ray	parallel
2AVH	GGGGTTTGGGG	X-ray	antiparallel
2GKU	TTGGGTTAGGGTTAGGGTTAGGGA	NMR	hybrid
2HY9	AAAGGGTTAGGGTTAGGGTTAGGGAA	NMR	hybrid
2JSM	TAGGGTTAGGGTTAGGGTTAGGG	NMR	hybrid
2JPZ	TTAGGGTTAGGGTTAGGGTTAGGGTT	NMR	hybrid
2JSL	TAGGGTTAGGGTTAGGGTTAGGGTT	NMR	hybrid
2KF8	GGGTTAGGGTTAGGGTTAGGGT	NMR	antiparallel
2KKA	AGGGTTAGGGTTAGGGTTAGGGT	NMR	antiparallel
2LD8	TAGGGTTAGGGTTAGGGTTAGGG	NMR	parallel
143D	AGGGTTAGGGTTAGGGTTAGGG	NMR	antiparallel
2L88*	GGGGCGGGGCGGGGCGGGGT	NMR	parallel
2L88*	GGGGCGGGGCGGGGCGGGGT	NMR	parallel
2L88*	GGGGCGGGGCGGGGCGGGGT	NMR	parallel
2KYP*	CGGGCGGGCGCTAGGGAGGGT	NMR	parallel
143D*	AGGGTTAGGGTTAGGGTTAGGG	NMR	antiparallel
2JSM*	TAGGGTTAGGGTTAGGGTTAGGG	NMR	hybrid
2JSL*	TAGGGTTAGGGTTAGGGTTAGGGTT	NMR	hybrid
2JSL*	TAGGGTTAGGGTTAGGGTTAGGGTT	NMR	hybrid

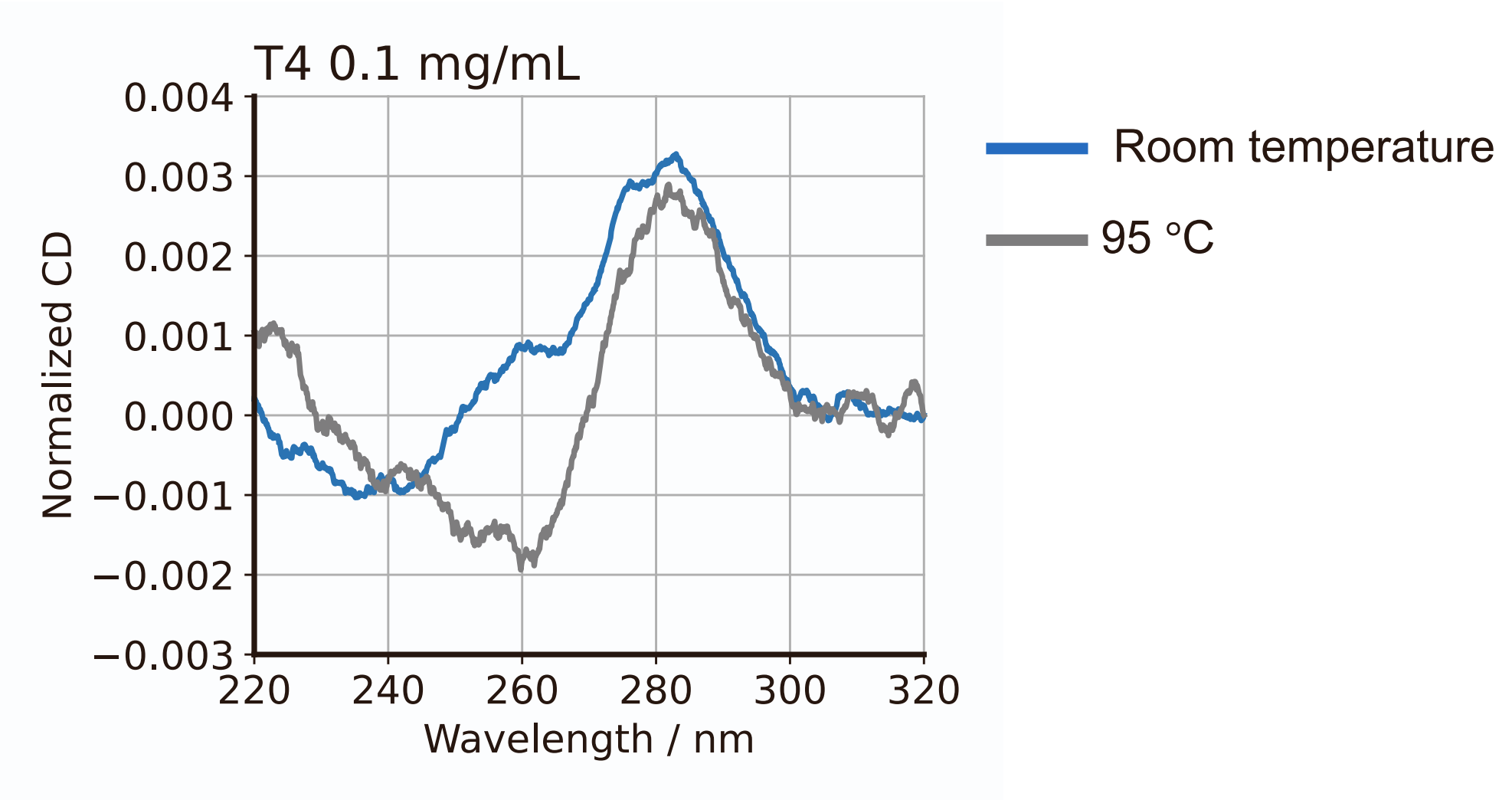

Figure S1. Comparison of CD spectra of T4 at 0.1 mg/mL recorded at room temperature and 95 °C. Blue, room temperature; gray, 95 °C.

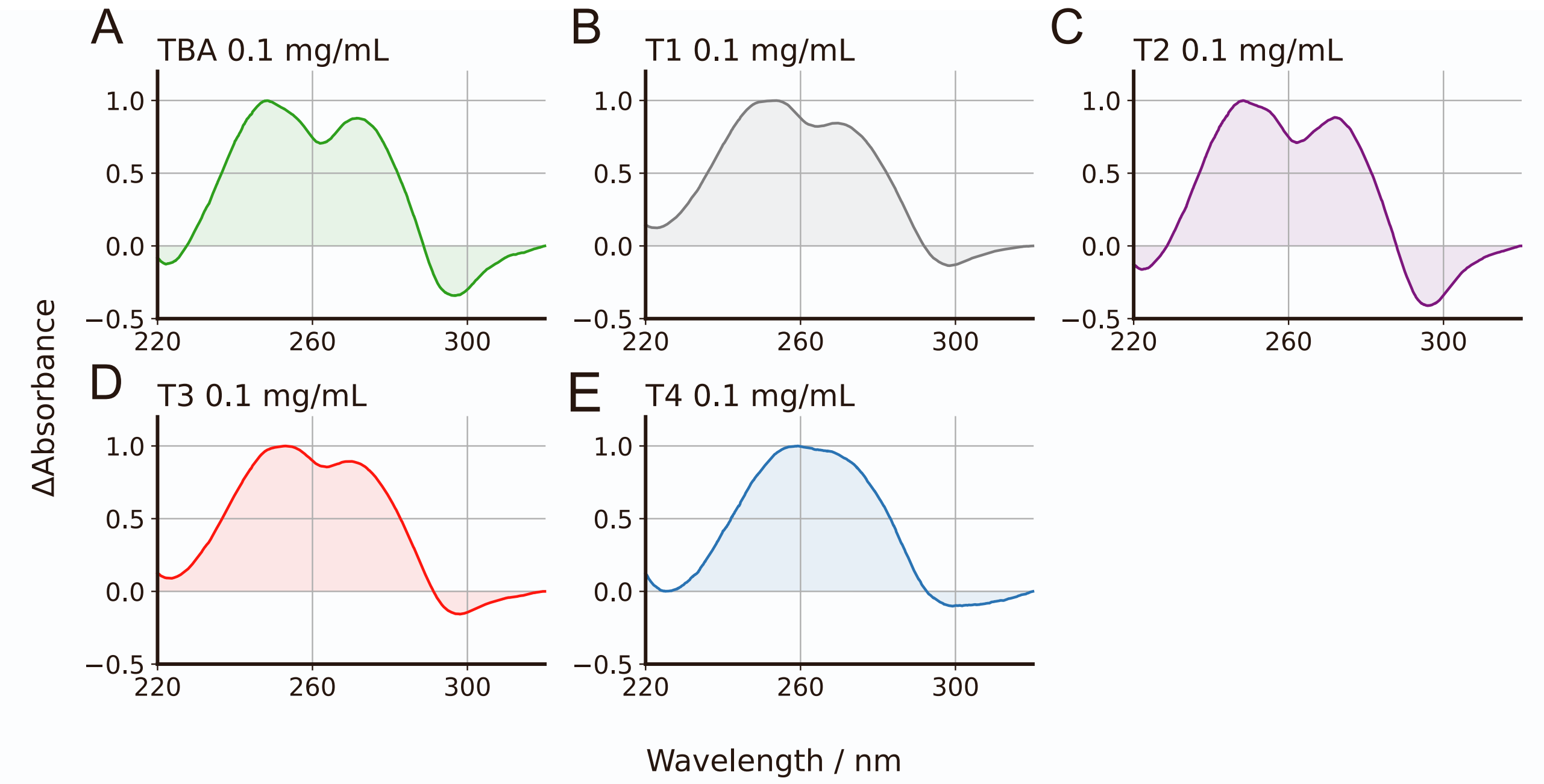

Figure S2. Thermal difference spectra (TDS) of TBA and modified TBAs at 0.1 mg/mL. Each TDS was obtained by subtracting the room-temperature absorption spectrum from the 95 °C absorption spectrum measured under the same concentration and path-length conditions. (A) TBA, (B) T1, (C) T2, (D) T3, and (E) T4.

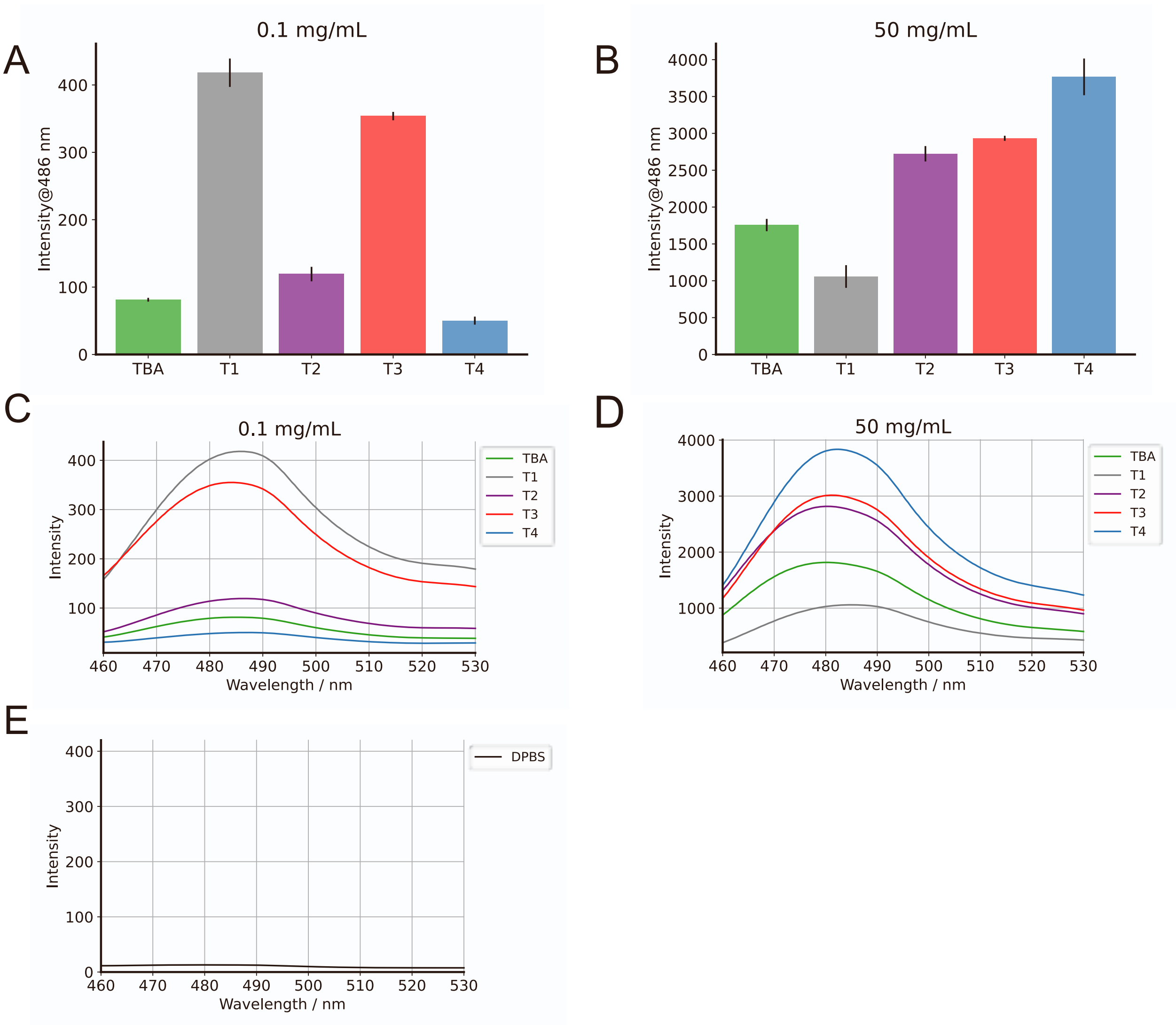

Figure S3. Comparison of ThT fluorescence. (A, C) Fluorescence intensity at 486 nm and corresponding fluorescence spectra at 0.1 mg/mL, respectively. Each spectrum represents the mean of three measurements. (B, D) Fluorescence intensity at 486 nm and corresponding fluorescence spectra at 50 mg/mL. (E) Fluorescence intensity for ThT in DPBS. The intensity at 486 nm and SDs was calculated from three measurements.

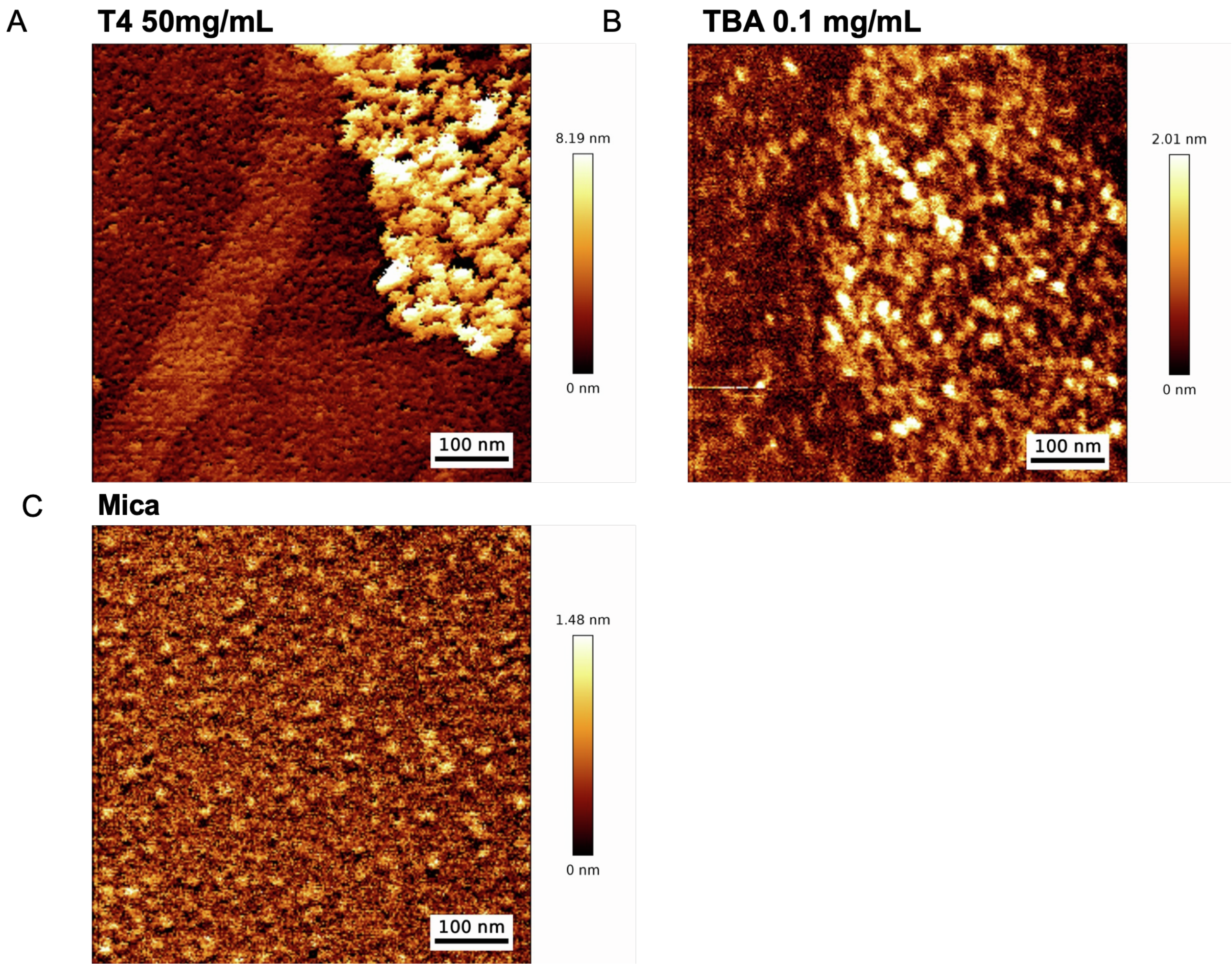

Figure S4. AFM morphological analysis.

AFM images for (A) 50 mg/mL T4, (B) 0.1 mg/mL TBA, and (C) mica surface.

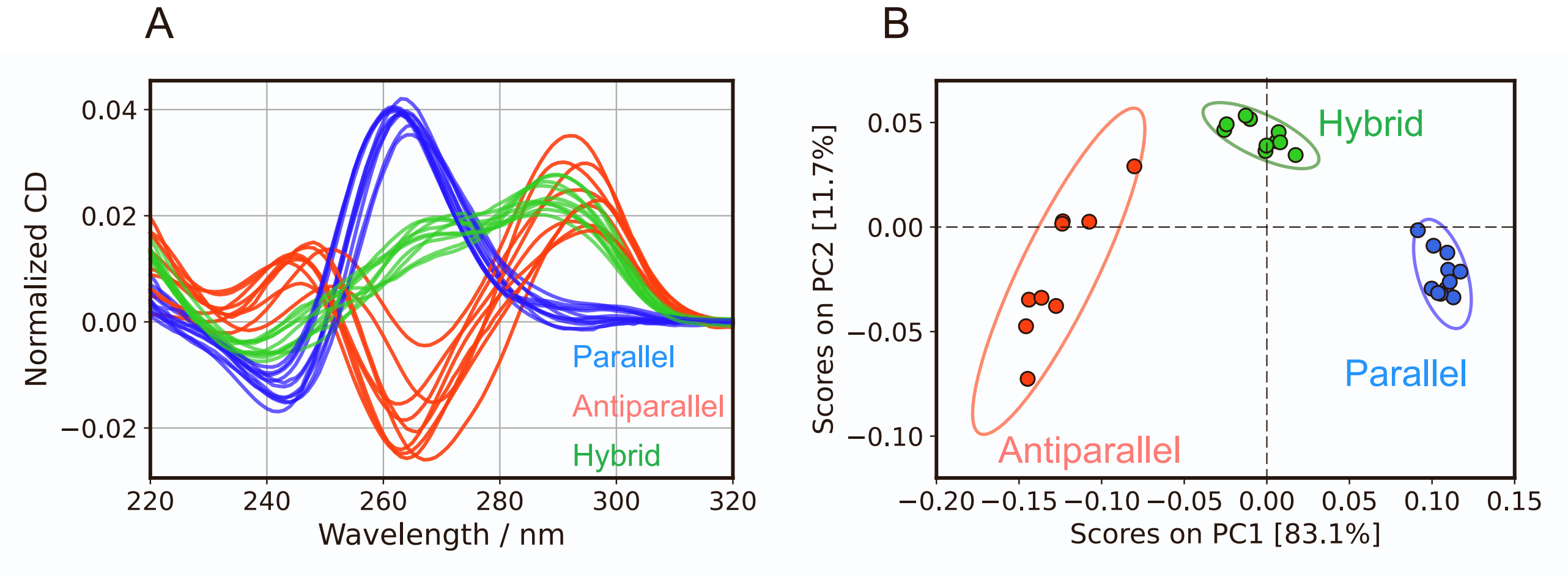

Figure S5. CD spectra of structurally characterized G4 reference sequences used for construction of (A) PCA model, and (B) resulting classification model. In panel B, PCA scores for individual reference spectra are plotted. Each cluster was defined based on hierarchical clustering of the reference spectra, and the 95% confidence ellipses are shown.

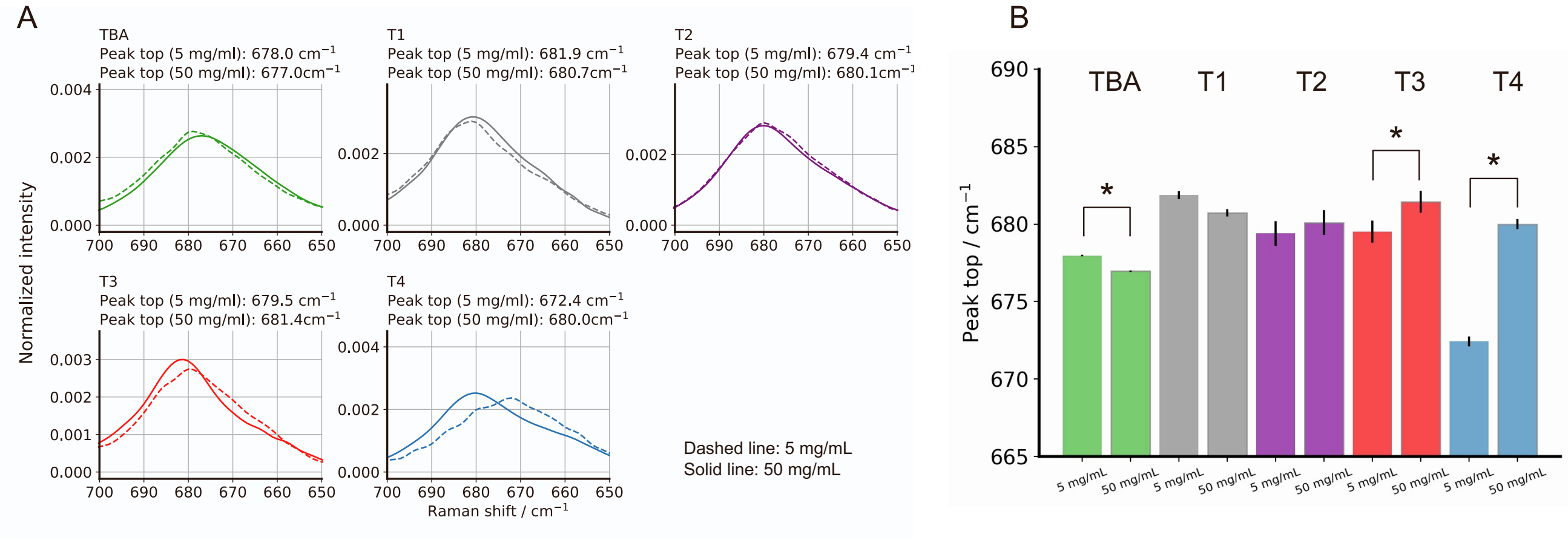

Figure S6. Raman spectra of TBA and modified TBAs in the vicinity of 680 cm-1. (A) Raman spectra of TBA and modified TBAs at 5 and 50 mg/mL. Each spectrum represents the mean of three measurements. Spectra recorded at 5 and 50 mg/mL are shown as dashed and solid lines, respectively. (B) Peak top positions calculated from the individual spectra. Asterisks indicate statistically significant differences ($p < 0.05$).

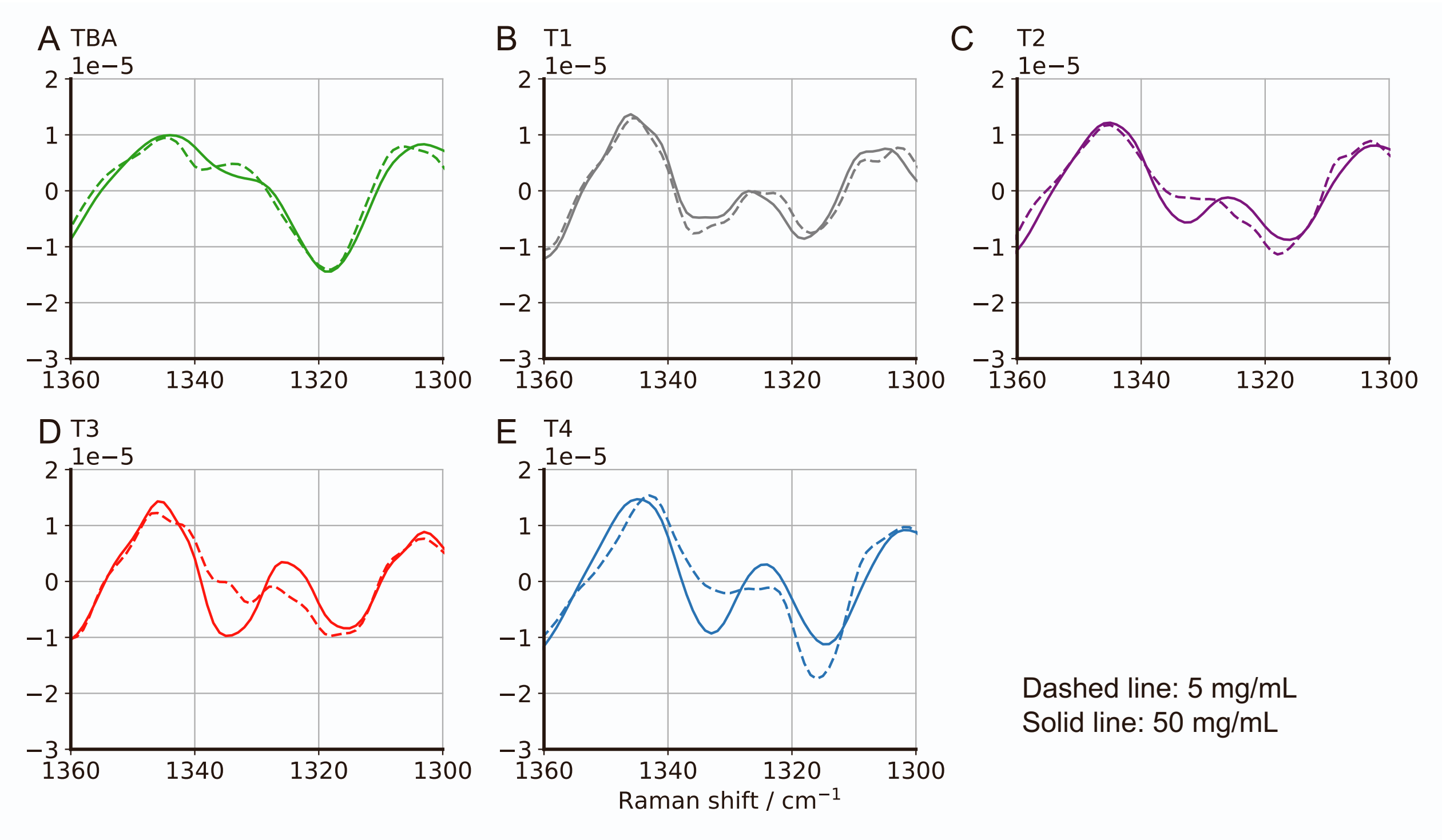

Figure S7. Raman spectra of (A) TBA, (B) T1, (C) T2, (D) T3, and (E) T4 in the vicinity of 1330 cm^{-1}. Spectra recorded at 5 and 50 mg/mL are shown as dashed and solid lines, respectively. Each spectrum represents the mean of three measurements. All spectra were subjected to second-derivative processing using the Savitzky–Golay method (window length, 15 cm^{-1}; polynomial order, 2).

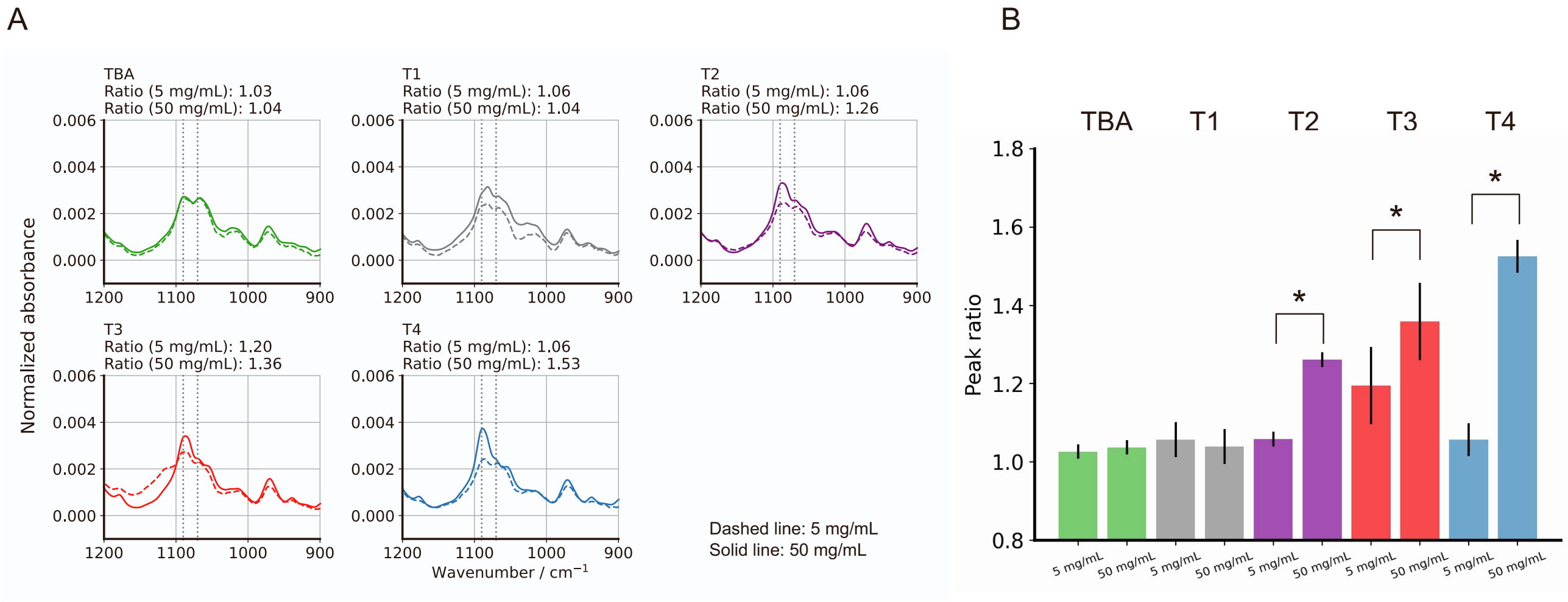

Figure S8. ATR FT-IR spectra of TBA and modified TBAs in the vicinity of 1100 cm^{-1}.

(A) ATR FT-IR spectra of TBA and modified TBAs at 5 and 50 mg/mL. Dashed and solid lines indicate spectra recorded at 5 and 50 mg/mL, respectively. Spectra of 5 mg/mL and 50 mg/mL represents the mean of five and three measurements, respectively. (B) Peak ratios calculated from the individual spectra. Asterisks indicate statistically significant differences ($p < 0.05$).

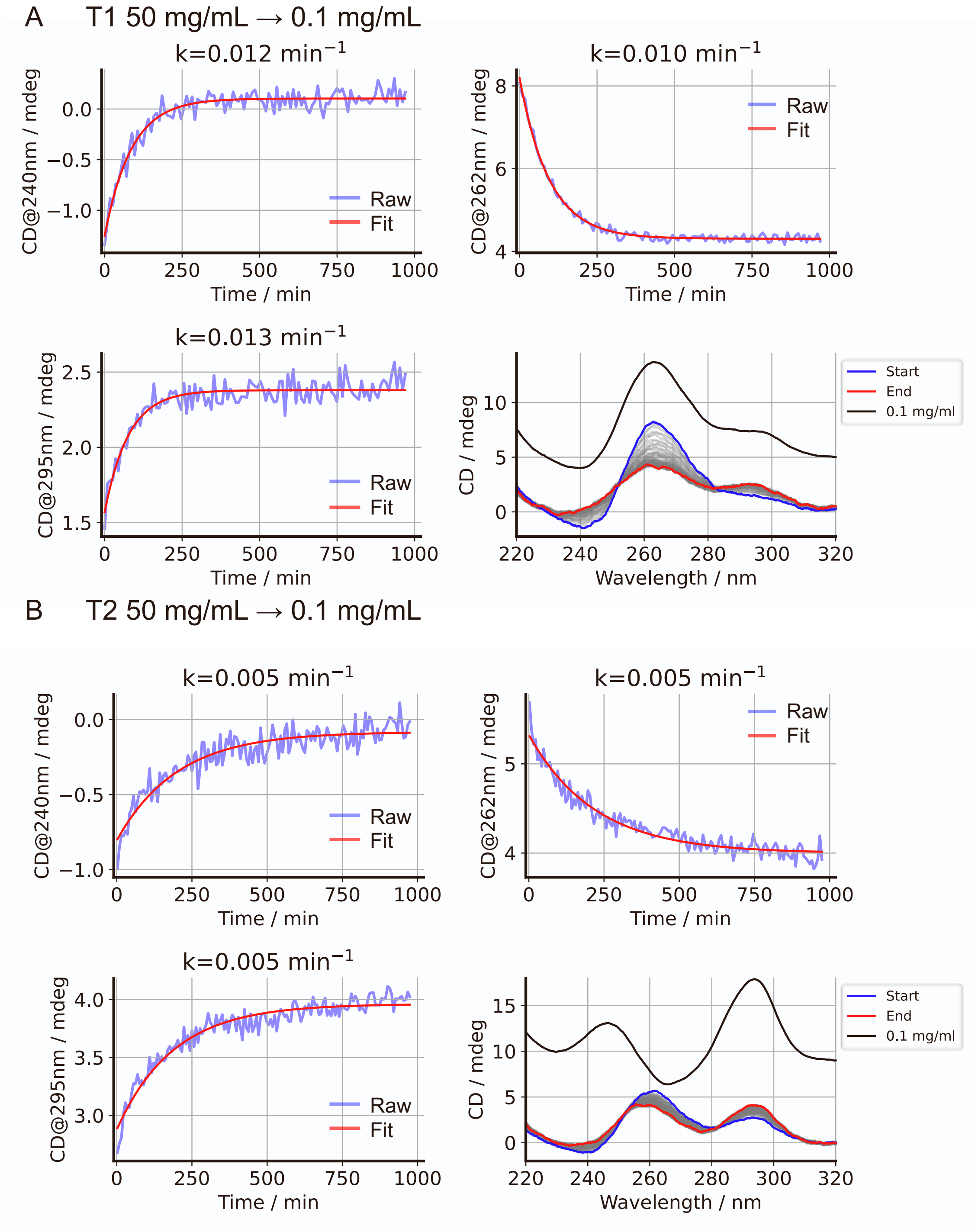

Figure S9. Time-dependent structural changes upon dilution.

(A) Representative time-dependent changes in CD intensity and (B) the corresponding time-dependent CD spectra used for the analysis for T1 and T2, respectively. Blue lines indicate the experimental CD values, and red lines indicate the fitting curves obtained using a first-order kinetic model.

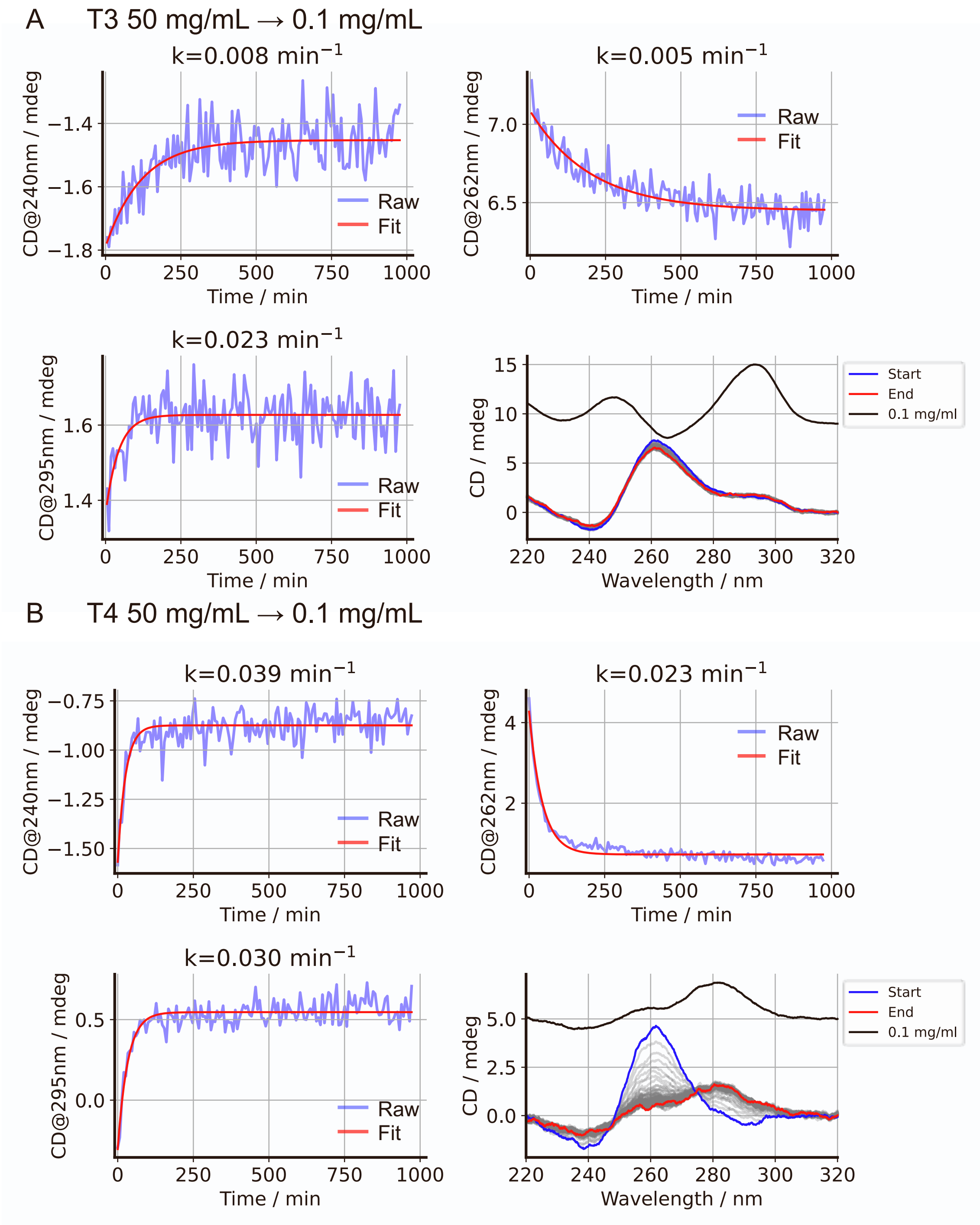

Figure S10. Time-dependent structural changes upon dilution.

(A) Representative time-dependent changes in CD intensity and (B) the corresponding time-dependent CD spectra used for the analysis for T3 and T4, respectively. Blue lines indicate the experimental CD values, and red lines indicate the fitting curves obtained using a first-order kinetic model.

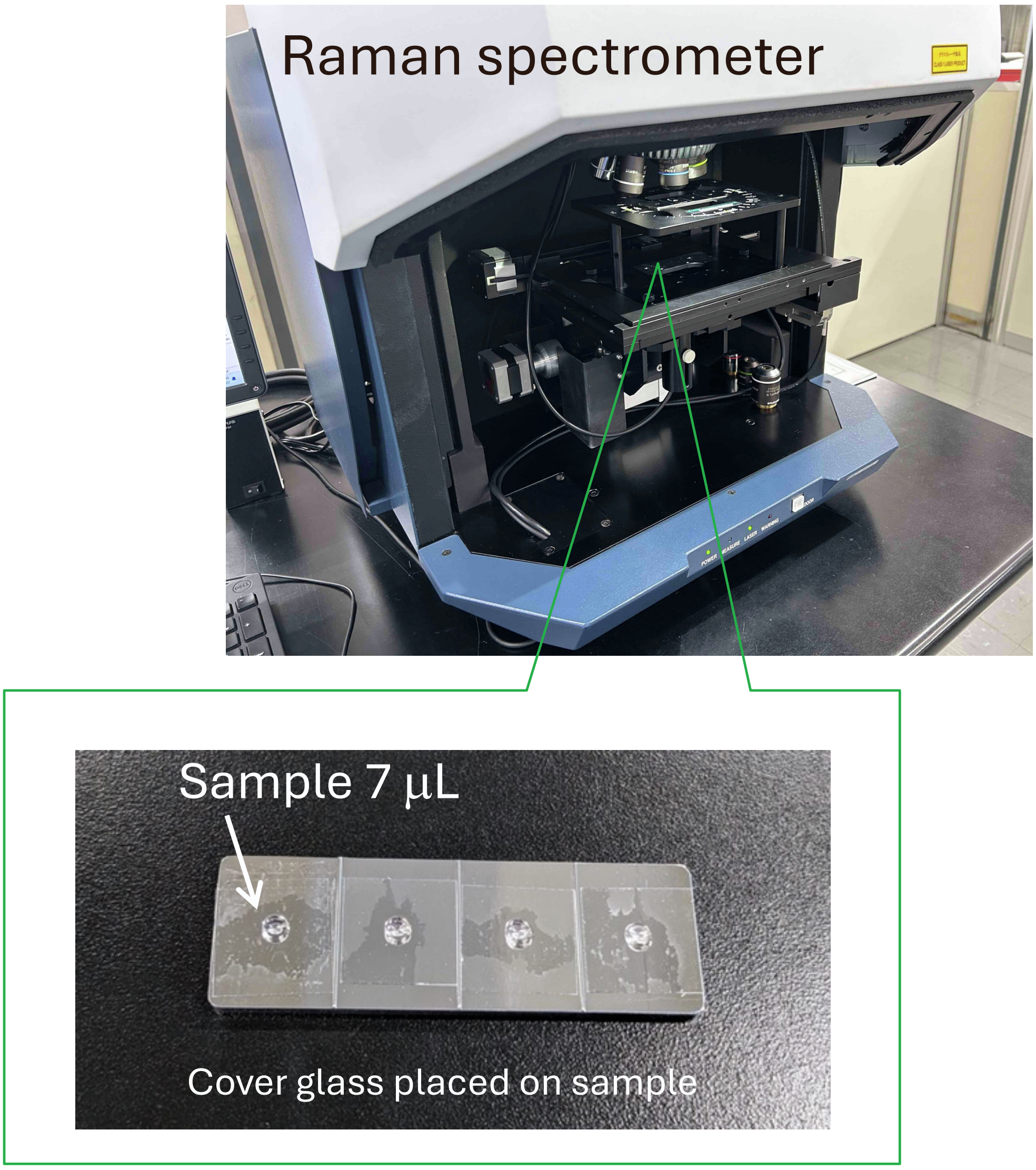

Figure S11. Photograph of custom-designed sample plate used for Raman spectroscopy measurements of TBA and modified TBAs.

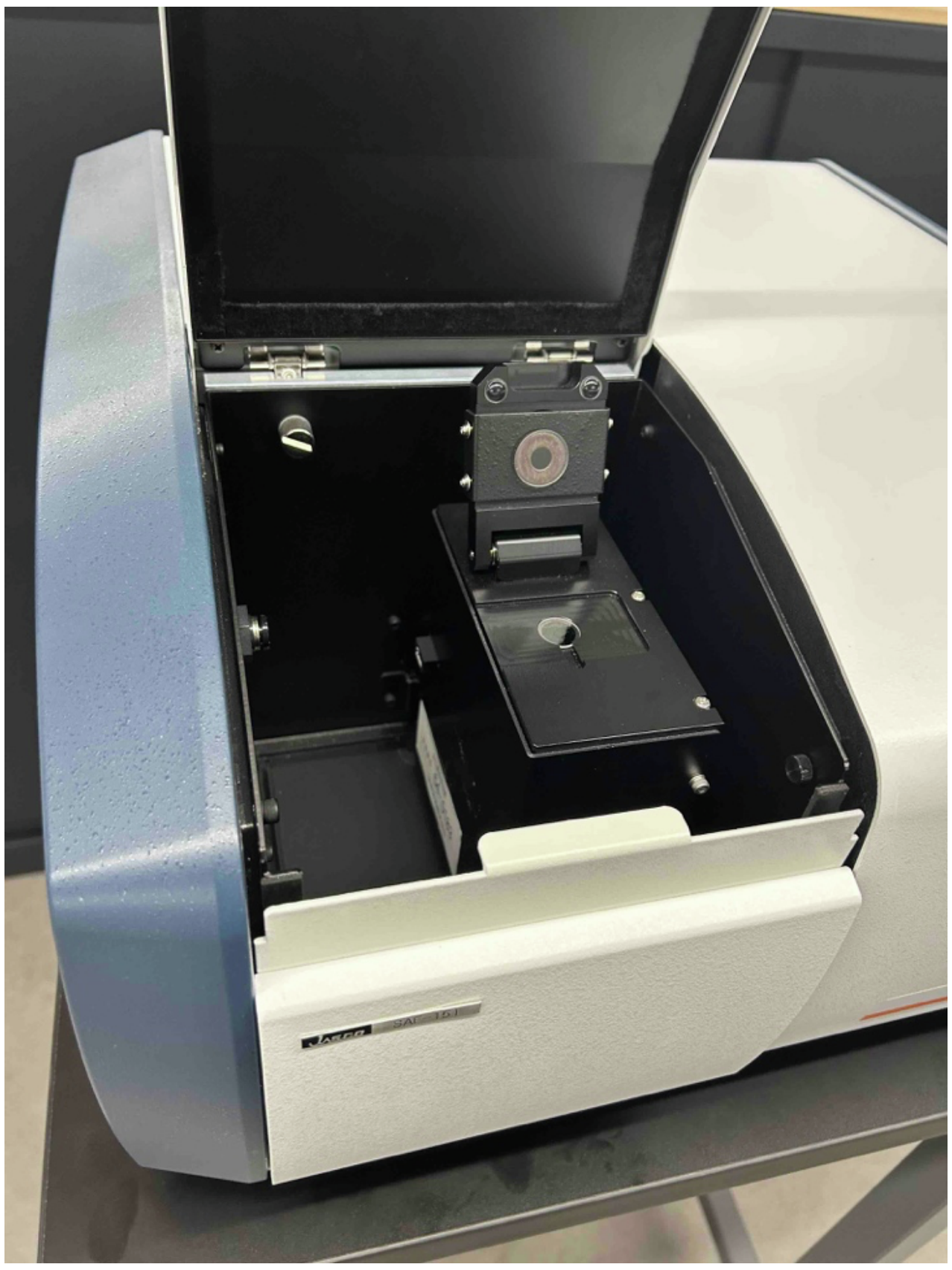

Figure S12. External view of the SAF-851 one-drop measurement unit. A 10 μL sample is sandwiched between dedicated quartz plates. Excitation light is directed onto the sample from below at an angle of 45°, and fluorescence emitted toward the lower side in a direction perpendicular to the quartz plate surface is detected.

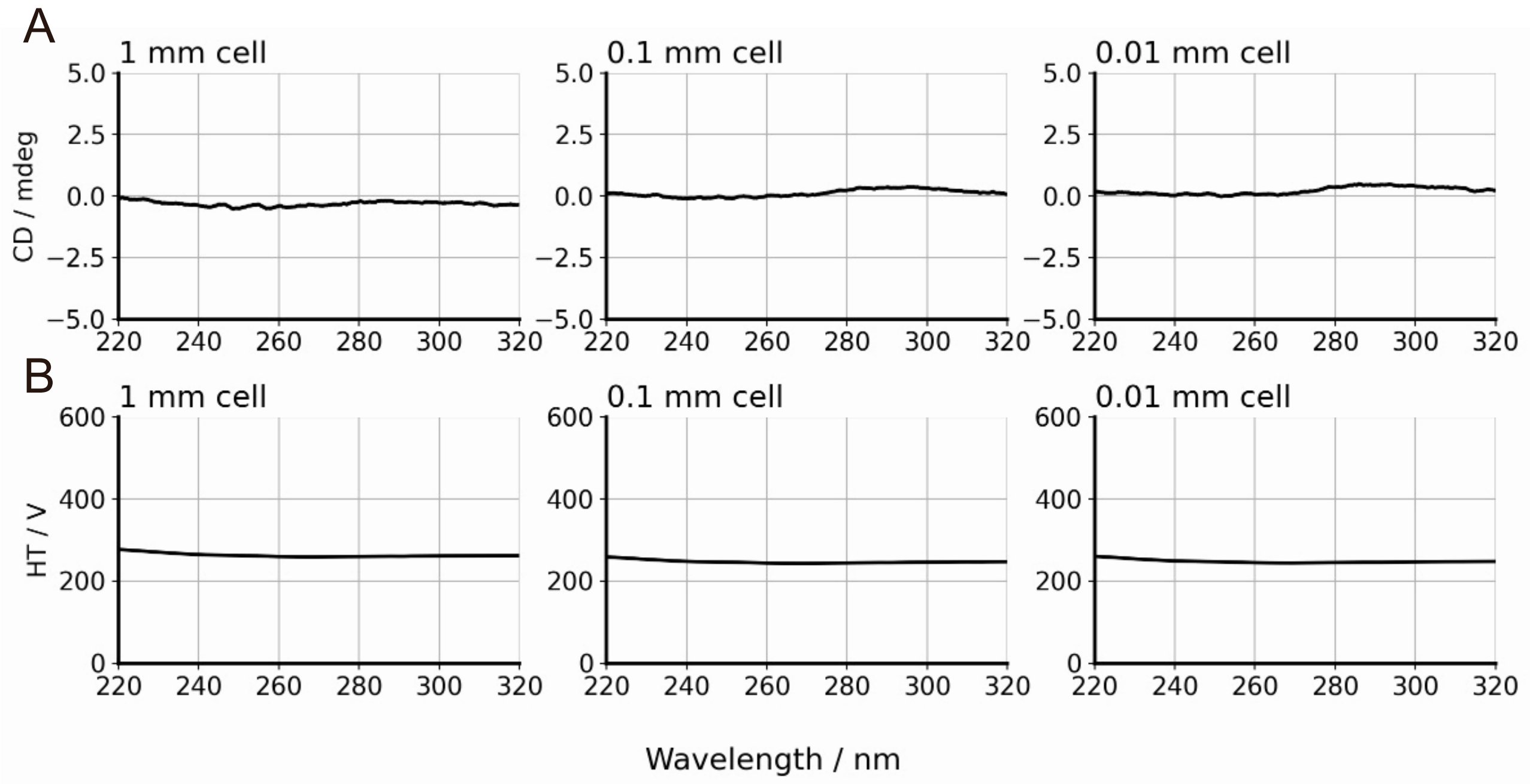

Figure S13. Buffer spectra measured using each cell.

(A) CD spectra and (B) HT voltage spectra.

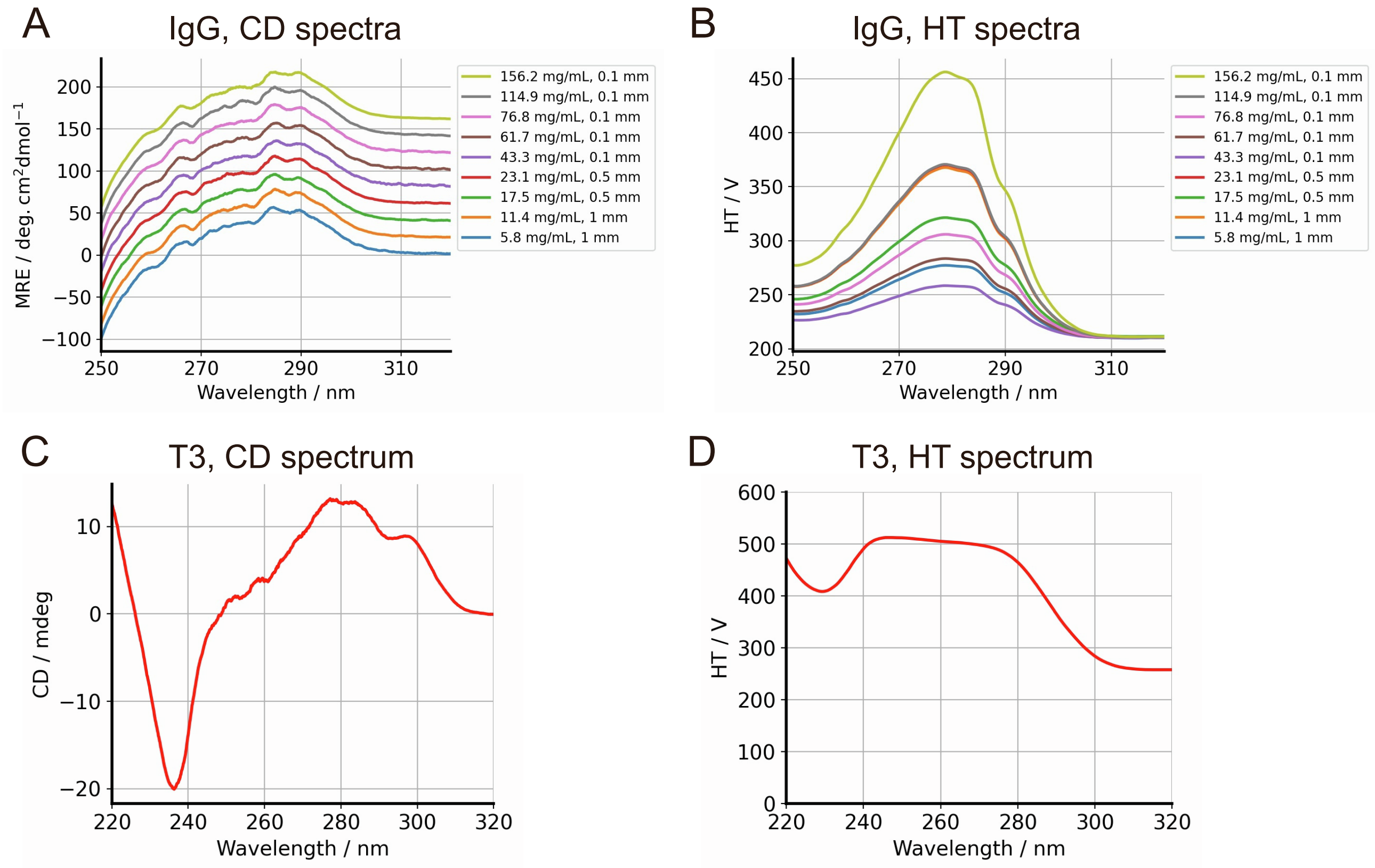

Figure S14. Effects of high-concentration samples on CD and HT voltage spectra.

(A, B) CD and HT voltage spectra of rabbit IgG in 20 mM citrate buffer at concentrations ranging from 5.8 to 156.2 mg/mL.

(C, D) CD and HT voltage spectra of 50 mg/mL T3 measured under conditions in which absorbance flattening was observed.
